# DRP-1-mediated apoptosis induces muscle degeneration in dystrophin mutants

**DOI:** 10.1038/s41598-018-25727-8

**Published:** 2018-05-09

**Authors:** Charlotte Scholtes, Stéphanie Bellemin, Edwige Martin, Maïté Carre-Pierrat, Bertrand Mollereau, Kathrin Gieseler, Ludivine Walter

**Affiliations:** 1 Laboratory of Biology and Modelling of the Cell, UMR5239 CNRS/Ecole Normale Supérieure de Lyon, UMS 3444 Biosciences Lyon Gerland, Universite de Lyon, Lyon, 69007 France; 2 NeuroMyoGene Institute (INMG), Universite Lyon 1, CNRS UMR 5310, INSERM U1217 Lyon 69008, France; 3 Biology of Caenorhabditis elegans facility, Universite Lyon 1, UMS3421 Lyon 69008, France

## Abstract

Mitochondria are double-membrane subcellular organelles with highly conserved metabolic functions including ATP production. Mitochondria shapes change continually through the combined actions of fission and fusion events rendering mitochondrial network very dynamic. Mitochondria are largely implicated in pathologies and mitochondrial dynamics is often disrupted upon muscle degeneration in various models. Currently, the exact roles of mitochondria in the molecular mechanisms that lead to muscle degeneration remain poorly understood. Here we report a role for DRP-1 in regulating apoptosis induced by dystrophin-dependent muscle degeneration. We found that: *(i)* dystrophin-dependent muscle degeneration was accompanied by a drastic increase in mitochondrial fragmentation that can be rescued by genetic manipulations of mitochondrial dynamics *(ii)* the loss of function of the fission gene *drp-1* or the overexpression of the fusion genes *eat-3* and *fzo-1* provoked a reduction of muscle degeneration and an improved mobility of dystrophin mutant worms *(iii)* the functions of DRP-1 in apoptosis and of others apoptosis executors are important for dystrophin-dependent muscle cell death *(iv)* DRP-1-mediated apoptosis is also likely to induce age-dependent loss of muscle cell. Collectively, our findings point toward a mechanism involving mitochondrial dynamics to respond to trigger(s) of muscle degeneration *via* apoptosis in *Caenorhabditis elegans*.

## Introduction

Mitochondria are double-membrane organelles that are crucial for many cellular processes such as aerobic ATP generation, calcium homeostasis, lipid biosynthesis or cell death^1^. Mitochondria are characterized by an important polymorphism of shapes due to permanent fusion and fission events that vary within a considered tissue^2^. Dynamin family GTPases regulates mitochondrial dynamics. In mammals, Mitofusin-1 and Mitofusin-2 (MFN1 and MFN2)^3^ allow for mitochondria outer membrane fusion whereas inner membrane fusion implicates Optic atrophy 1 (OPA1)^4^. Fission machinery of the outer membrane is composed of Dynamin-related protein1 (DRP1) and Dynamin-2 (DYN2)^5,6^. In *Caenorhabiditis elegans* (*C. elegans*), DRP-1 is also required for mitochondrial fission^7^ whereas EAT-3 (OPA1 homolog in mammals) and FZO-1 (MFN1 homolog in mammals) control inner and outer mitochondrial membrane fusion, respectively^8^. Mitochondrial fusion is essential for normal mitochondrial activity notably by diluting damaged mitochondrial DNA, lipids or proteins^9,10^. In mammals, mitochondrial fission is key for mitophagy to eliminate damaged mitochondria^11,12^. Also, fragmented mitochondria often appear in the early steps of cell death associated with permeabilization of the mitochondrial outer membrane, which induces the release of inter membrane space-stored proapoptotic factors, such as cytochrome *c*, Apoptosis-Inducing Factor (AIF) or Endonuclease G (EndoG)^13–16^. In *C. elegans*, fragmentation of mitochondria during apoptosis has been reported but the exact roles of mitochondria in cell death remain largely unknown^17^. Whether mitochondrial fusion is directly implicated in apoptosis is still under debate. In mammals, hyperfusion allows for survival under numerous stresses including nutrient deprivation^18–21^. Furthermore, the fusion proteins MFN1 and MFN2 seem to directly regulate apoptosis suggesting that longer mitochondria are more supportive for cell survival than fragmented mitochondria^22,23^. Interestingly, molecular links exist between mitochondrial fusion and apoptosis^24–26^.

Importantly, the main actors of mitochondrial fission and fusion have alternative functions separate from mitochondrial dynamics. It is well established that DRP-1 participates in peroximal division^27,28^ and in the maintenance of the morphology and the function of the Endoplasmic Reticulum (ER)^29,30^. In *C. elegans*, DRP-1 can contribute to apoptotic processes independently from mitochondrial fission^31^. Furthermore, both DRP-1 and OPA1 allow for mitochondrial cristae remodeling separately from mitochondrial dynamics^32–34^. The alternative functions of OPA1 also include lipolysis regulation^35^. The pro-fusion protein MFN2 is an essential component of the contact sites between mitochondria and ER and contributes to mitochondrial Ca^2+^ uptake and ER morphology maintenance^36^. Additionally, levels of MFN2 are regulated by ER stress and can affect the ER-Unfolded Protein Response (ER-UPR) and in turn, cell death^37,38^. Furthermore, axonal mitochondrial transport requires MFN2 but not mitochondrial fusion^39^.

Strength and function of muscle tissues are highly dependent on the energy produced by mitochondria. Compromised muscle tissues, due to deterioration and loss of muscle cells, and leading to muscle weakness, are hallmarks of muscle degeneration. The primary causes of muscle degeneration are various. For instance, it can be due to numerous genetic mutations causing muscular dystrophies. Muscle degeneration can also be caused by aging processes, and is called sarcopenia. To study muscle degeneration, we used the *dys-1(cx18);hlh-1(cc561ts) C. elegans* mutant, which exhibits progressive dystrophin-dependent muscle degeneration reflected by muscle weakness and dramatic loss of muscle cells^40^. DYS-1 is the homolog of dystrophin in mammals and mutations in this gene cause Duchenne Muscular Dystrophy in Human^41^. HLH-1 is a homolog of Myogenic Differentiation 1 (MyoD), a muscle transcription factor^42^. Despite the phylogenetic distance between mammals and *C. elegans*, the nematode presents striated body wall muscle similar to vertebrate skeletal muscle in terms of function and structural components; however, the overall architecture is less complex^43,44^. Mitochondrial fragmentation increases in muscle cells during dystrophin-dependent muscle degeneration in various animal models including nematode^45^, zebrafish^45^, mouse, which exhibits abnormalities of mitochondrial morphology and density as well as an up-regulation of DRP-1 levels^46,47^, dog^48^ and in biopsies from Duchenne Muscular Dystrophy patients^49^. Fragmentation of mitochondria is also observed during muscle aging in nematode^50^, fly^51^ and mouse^52^.

We hypothesized that genetic manipulations of the main actors of mitochondrial dynamics can improve muscle health. We found that decreasing mitochondrial fission or increasing mitochondrial fusion can partially rescue mitochondrial dynamics, reduce muscle degeneration and improve the mobility of *dys-1(cx18);hlh-1(cc51ts)* mutant worms. Conversely, increased mitochondrial fusion can enhance the muscle degeneration phenotype of dystrophic nematodes. Interestingly, we showed that DRP-1 functions mainly in the apoptosis pathway downstream of the caspase 3 to impact muscle degeneration and that others apoptotic executors are important for muscle health. Finally, apoptosis induced by DRP-1 seems also to be implicated in aging-dependent muscle loss. Collectively, our results point toward a novel mechanism by which, DRP-1-mediated apoptosis provokes muscle degeneration.

## Results

### Mitochondrial fragmentation increases upon dystrophin-deficiency

**Figure 1:**
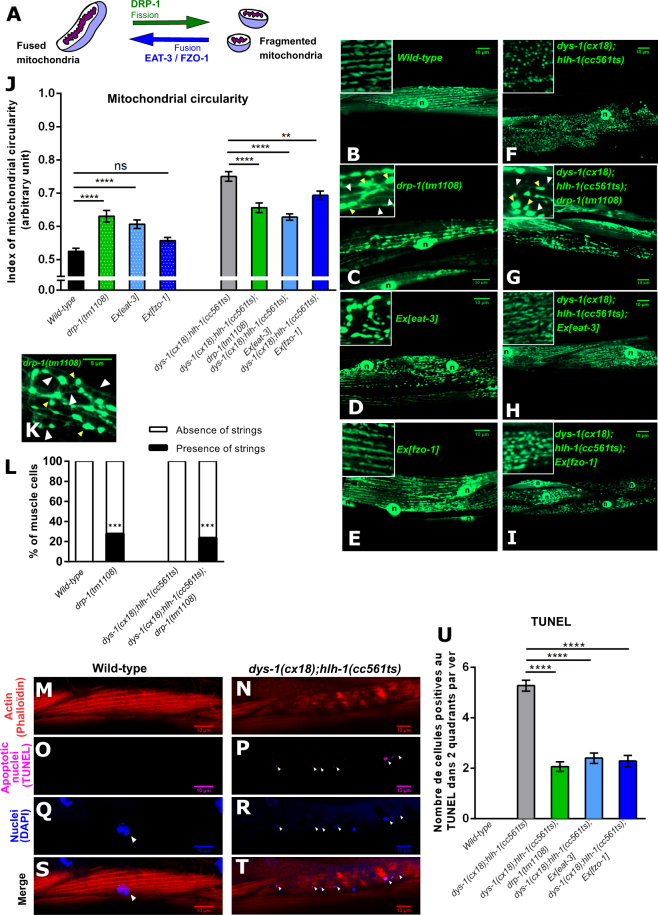
DRP-1 induces mitochondrial fragmentation and apoptosis upon dystrophin-dependent muscle degeneration. (**A**) Schematic representation of the main mitochondrial dynamics actors in *C. elegans*. Representative confocal images of the mitochondrial network organization visualized with the *ccIs4251* transgene in *C. elegans* body wall muscle cells of: (**B**) wild-type worms; (**C**) *drp-1(tm1108*) mutant worms; (**D**) worms overexpressing *eat-3;* (**E**) worms overexpressing *fzo-1;* (**F**) *dys-1(cx18);hlh-1(cc561ts)* mutant worms; (**G**) *dys-1(cx18);hlh-1(cc561ts)*;*drp-1(tm1108)* mutant worms; (**H**) *dys-1(cx18);hlh-1(cc561ts)* overexpressing *eat-3* mutant worms and (**I**) *dys-1(cx18);hlh-1(cc561ts)* overexpressing *fzo-1* mutant worms. Scale bar 10 μm (x630). n indicates nuclei. Yellow arrowheads indicate bubble-like shape mitochondria. White arrowheads indicate mitochondria connecting strings. (**J**) Quantification of mitochondrial circularity in each of the indicated strains (n = 29 worms at least). (**K**) Representative confocal image of thin mitochondrial filaments in between blebs of mitochondria in the *drp-1(tm1108)* mutant worms. Scale bar 5 μm (x630). Yellow arrowheads indicate bubble-like shape mitochondria. White arrowheads indicate mitochondria connecting strings. (**L**) Percentage of muscle cells exhibiting strings in between blebs of mitochondria in each of the indicated strains (n = 54 worms at least). Chi-square test. ***p < 0.001. Representative confocal images: of actin network by phalloïdin staining in (**M**) wild-type worms and (**N**) *dys-1(cx18);hlh-1(cc561ts)* mutant worms; of apoptotic nuclei revealed by TUNEL staining in (**O**) wild-type worms and (**P**) *dys-1(cx18);hlh-1(cc561ts)* mutant worms; of nuclei of muscle cells stained by DAPI in (**Q**) wild-type worms and (**R**) *dys-1(cx18);hlh-1(cc561ts)* mutant worms and of the merge of the phalloidin, TUNEL and DAPI staining in (**S**) wild-type worms and (**T**) in *dys-1(cx18);hlh-1(cc561ts)* mutant worms. Scale bar 10 μm. n indicates nuclei. White arrowheads indicate colocalization of DAPI and TUNEL staining. (x630). (**U**) Quantification of muscle TUNEL positive cells in each of the indicated strains (n = 55 worms at least). All the experiments were performed on L4 + 3 day-old worms. One-way ANOVA, Tukey’s multiple comparisons test. Data represent the mean obtained by pooling at least three independent assays. Errors bars represent SEM (Standard Error to the Mean) **p < 0.01 ****p < 0.0001 ns. indicates that the mean is not statistically significantly different from the mean obtained in the control condition.

Mitochondrial shapes undergo continual changes through the combined actions of fission and fusion events rendering mitochondrial network very dynamic. In *C. elegans*, fission is mediated by DRP-1^7^, inner membrane fusion by EAT-3 (OPA1 homolog)^53^ and outer membrane fusion by FZO-1 (MFN1 homolog)^8^ (Fig. 1A). As *C. elegans* is a transparent organism, it is possible to visualize and evaluate mitochondrial network shape of muscle cells in lived worms^54^. In order to visualize mitochondrial morphologies, we used the *ccIs4251* transgene, a genomic integrated GFP construction addressed specifically to the mitochondrial matrix and nuclei of the *C. elegans* body wall muscle cells (Fig. 1B–I)^55^. As previously published by Yang *et al*.^54^, the index of circularity of individual mitochondrion (a mathematical estimation of circular shapes) was calculated for each of the tested conditions to evaluate mitochondrial dynamics. A value of 1 for the index of mitochondria circularity means that mitochondria are perfectly circular and a value of 0 means that mitochondria are perfectly flat. In wild-type *C. elegans* muscle cells, tubular and circular mitochondria were in equal proportions (Fig. 1B) as reflected by a mitochondrial circularity index of around 0,5 (Fig. 1J)^54^. *dys-1(cx18);hlh-1(cc561ts)* mutant worms, which present progressive muscle degeneration, exhibited mitochondrial dynamics perturbations with more fragmented mitochondria in muscle cells than wild-type worms (Fig. 1B,F) as reflected by a higher index of mitochondria circularity compared to that of wild-type worms (0,75 +/− 0,014 in *dys-1(cx18);hlh-1(cc561ts)* mutant *vs* 0,52 +/− 0,009 in wild-type worms) (Fig. 1J). By comparing mitochondrial aspect ratio (major axis/minor axis of each mitochondrion), mitochondrial elongation (1-(minor axis/major axis of each mitochondrion) and mitochondrial size, we also found that *dys-1(cx18);hlh-1(cc561ts)* mutant worms presented shorter and smaller mitochondria than that of wild-type worms (Fig. S1A–D). Furthermore, dystrophic worms exhibited a less connected mitochondrial network than wild-type animals (Fig. S1D).

In order to decrease mitochondrial fission, we introduced the *drp-1(tm1108)* null mutation^31^ in the *dys-1(cx18);hlh-1(cc561ts)* mutant worms. Mitochondria of *drp-1(tm1108)* mutant worms formed bubbles-like shape (Fig. 1C), as previously published^7,31,54,56^, presumably because fission of the mitochondrial inner membrane still occurs in absence of DRP-1^7^ suggesting that it is possible to separate outer and inner membrane fissions in *C. elegans*. Accordingly, mitochondria of *drp-1(tm1108)* mutant worms exhibited increased circularity and size but were shorter compared to that of wild-type worms (Figs 1J and S1A–C)^54^. In addition, altered DRP-1 expression provoked the apparition of long and thin mitochondrial filaments in between the mitochondrial blebs (Fig. 1C,K) in ~25% of the cells (Fig. 1L)^7^. Mitochondria of *dys-1(cx18);hlh-1(cc561ts)*;*drp-1(tm1108)* mutant worms appeared less circular, longer and with the occasional presence of interconnecting mitochondrial strings when compared to those of *dys-1(cx18);hlh-1(cc561ts)* mutant worms (Figs 1G,J,L and S1A–D), reflecting a rescue of the mitochondrial fragmentation phenotype induced by dystrophin deficiency. Moreover, the index of mitochondrial circularity of *dys-1(cx18);hlh-1(cc561ts)* mutant worms was significantly decreased in absence of DRP-1 (0,75 +/− 0,014 in *dys-1(cx18);hlh-1(cc561ts)* mutant *vs* 0,65 +/− 0,014 in *dys-1(cx18);hlh-1(cc561ts)*;*drp-1(tm1108)* mutant worms) (Fig. 1J). Similar results were obtained when *drp-1* was knock-downed by RNAi (Fig. S1E–I)^45^. To ensure that the machinery of mitochondrial dynamics was responsible for changes in mitochondrial morphologies upon dystrophin-dependent muscle degeneration, we overexpressed the fusion promoting genes *eat-3* or *fzo-1*. Unexpectedly, in wild-type worms, overexpression of *eat-3* increased mitochondrial fragmentation (Fig. 1D) while *fzo-1* overexpression had no statistically significant effects on mitochondrial morphology (Fig. 1E). Accordingly, mitochondrial circularity indexes were increased upon *eat-3* overexpression and unmodified upon *fzo-1* overexpression compared to that of wild-type worms (Fig. 1J). These surprising effects of *eat-3* and *fzo-1* overexpression have already been observed in *C. elegans* embryos suggesting that overexpressing fusion machinery in a wild-type background is not sufficient to cause mitochondrial fusion^25^. Furthermore, EAT-3 may act in both fusion and fission in *C. elegans* similarly to what was shown in mammals for OPA1^57^. Importantly, both *eat-3* and *fzo-1* overexpression provoked an apparent increased mitochondrial fusion in *dys-1(cx18);hlh-1(cc561ts)* mutant worms accompanied by a decreased mitochondrial circularity index when compared to that of *dys-1(cx18);hlh-1(cc561ts)* mutant worms (0,62 +/− 0,009 in *dys-1(cx18);hlh-1(cc561ts);Ex[eat-3*] mutant and 0,69 +/− 0,013 in *dys-*1*(cx18);hlh-1(cc561ts);Ex[fzo-1]* mutant *vs* 0,75 +/− 0,014 in *dys-1(cx18);hlh-1(cc561ts)* mutant worms) (Fig. 1H–J). Overall, absence of the fission protein DRP-1 or increased levels of the fusion proteins EAT-3 and FZO-1 can partially rescue dystrophin-dependent mitochondrial fragmentation in muscles cells. Our data demonstrate that mitochondrial dynamics and its principal key players are impacted by the loss of dystrophin in *C. elegans*.

### DRP-1 induces dystrophin-dependent apoptosis

We next tested whether mitochondrial dynamics could impact muscle cell death in *dys-1(cx18);hlh-1(cc561ts)* mutant worms. To that extent, we performed TUNEL (Terminal deoxynucleotidyl transferase dUTP nick end labeling) assays along with actin staining on wild-type and *dys-1(cx18);hlh-1(cc561ts)* mutant worms. TUNEL stains DNA fragments resulting from apoptosis-induced DNA fragmentation^58^. In wild-type worms, the actin network in muscle cells was nicely and regularly organized in filaments along the diamond-shaped cell with the nuclei, centered along the anterior-posterior axis, showing no staining by TUNEL. By contrast, in *dys-1(cx18);hlh-1(cc561ts)* mutant worms, muscle cells often presented abnormal phenotypes with actin network perturbations (*i.e* actin filaments form wavelets and/or aggregates) and with fragments of nuclei displayed over the entire cell and stained by TUNEL (Fig. 1M–T). The number of TUNEL positives muscle cells decreased in *dys-1(cx18);hlh-1(cc561ts)* mutant worms in absence of the pro-fission protein DRP-1 or when *eat-3* and *fzo-1* were overexpressed (2.07 +/− 0.19 TUNEL positive cells in *dys-1(cx18);hlh-1(cc561ts)*;*drp-1(tm1108)* mutant *vs* 2,4 +/− 0,20 in *dys-1(cx18);hlh-1(cc561ts);Ex[eat-3]* mutant *vs* 2,3 +/− 0,21 in *dys-1(cx18);hlh-1(cc561ts);Ex[fzo-1]* mutant *vs* 5,28 +/− 0,26 in *dys-1(cx18);hlh-1(cc561ts)* mutant worms) (Fig. 1U). These data were confirmed using two independent lines overexpressing *eat-3* and *fzo-1* (Fig. S2). Hence, genetically decreased mitochondrial fission or increased mitochondrial fusion can both reduce apoptosis induced by dystrophin deficiency in muscle.

### DRP-1 induces dystrophin-dependent muscle degeneration and locomotion defects

**Figure 2:**
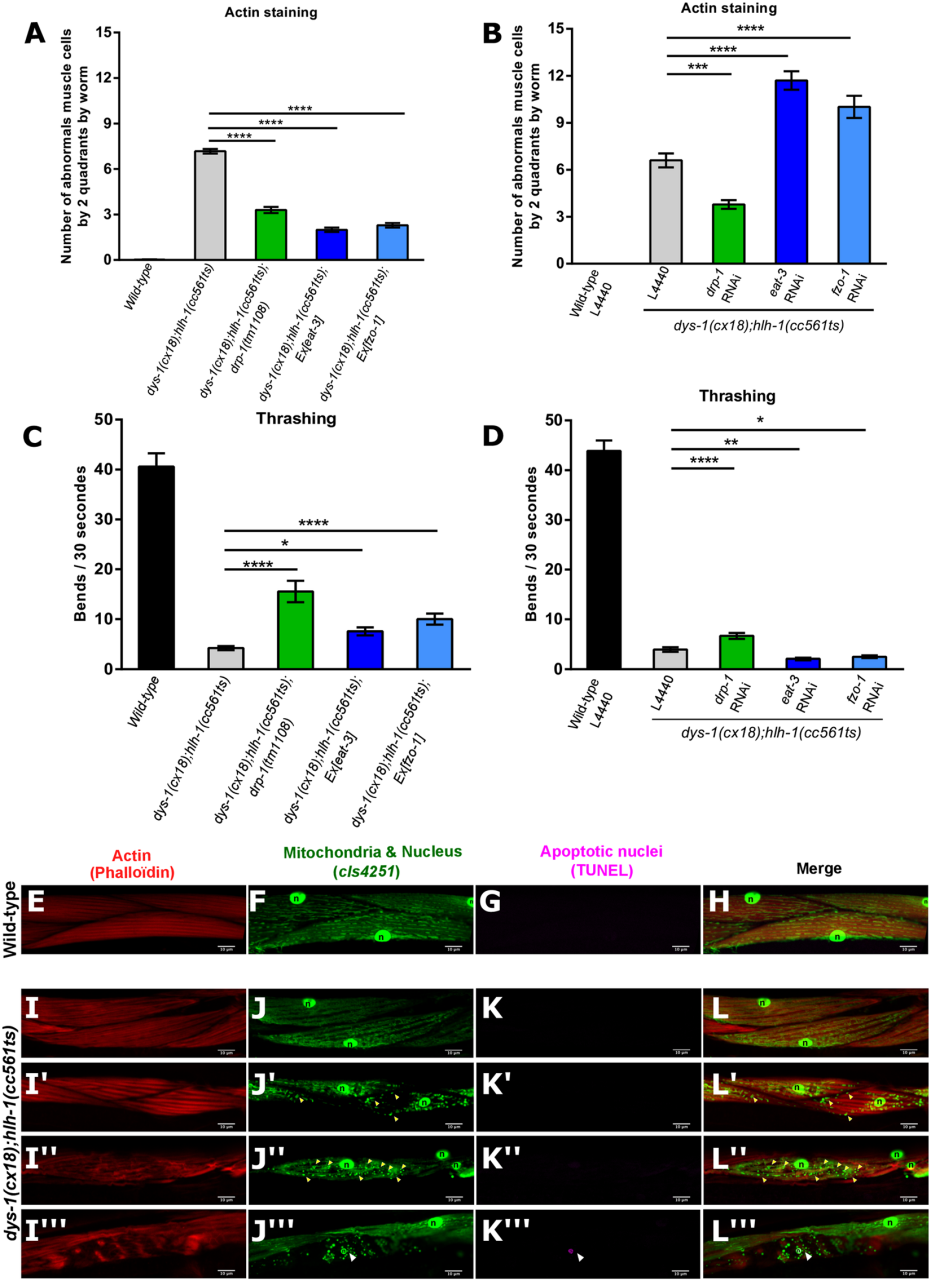
DRP-1 induces dystrophin-dependent muscle degeneration and locomotion defects. (**A**) Number of abnormal *C. elegans* body wall muscle cells by two quadrants quantified by phalloïdin staining in wild-type worms, *dys-1(cx18);hlh-1(cc561ts)* mutant worms, or *dys-1(cx18);hlh-1(cc561ts)* mutant worms with *drp-1(tm1108)* mutation or overexpression of *eat-3* or *fzo-1* (n = 70 worms at least). (**B**) Number of abnormal *C. elegans* body wall muscle cells by two quadrants quantified by phalloïdin staining in *dys-1(cx18);hlh-1(cc561ts)* mutant worms fed either with the empty vector L4440 or with RNAi constructs targeting *drp-1* or *eat-3* or *fzo-1* (n = 60 worms at least). (**C**) Quantification of worm trashing in wild-type worms, *dys-1(cx18);hlh-1(cc561ts)* mutant worms or *dys-1(cx18);hlh-1(cc561ts)* mutant worms with *drp-1(tm1108)* mutation or overexpression of *eat-3* or *fzo-1* (n = 25 worms at least). (**D**) Quantification of worm trashing in *dys-1(cx18);hlh-1(cc561ts)* mutant worms fed with the empty vector L4440 or with *drp-1* RNAi or *eat-3* RNAi or *fzo-1* RNAi (n = 100 worms at least). All the experiments were performed on L4 + 3 day-old worms. One-way ANOVA, Tukey’s multiple comparisons test. Data represent the mean obtained by pooling at least three independent assays. Errors bars represent SEM *p < 0.05 **p < 0.01 ***p < 0.001 ****p < 0.0001 ns. indicates that the mean is not statistically significantly different from the mean obtained in the control condition. Representative confocal images of muscles in wild-type worms of: (**E**) actin network by phalloïdin staining; (**F**) mitochondrial network and nuclei (n) organization visualized with the *ccIs4251* transgene in *C. elegans* body wall muscle cells; (**G**) apoptotic nuclei visualized by TUNEL staining and (**H**) the merge of the phalloidin, mitochondrial and TUNEL staining. (**I-** L”’) Representative confocal images of body wall muscle cells of *dys-1(cx18);hlh-1(cc561ts)* mutant worms presenting different degrees of muscle degeneration. (**I-**I”’) actin network by phalloïdin staining; (**J-**J”’) mitochondrial network visualized with the *ccIs4251* transgene in *C. elegans* body wall muscle cells; (**K-**K”’) apoptotic nuclei visualized by TUNEL staining; (**L-**L”’) merge of the phalloidin, mitochondrial and TUNEL staining. All the experiments were performed in L4 + 3 day-old worms. Yellow arrows indicate circular mitochondria. White arrow indicates apoptotic nuclei. Scale bar 10 μm. n indicates nuclei. (x630).

To analyze the impact of mitochondrial dynamics manipulation on muscle health in *dys-1(cx18);hlh-1(cc561ts)* mutant worms, we further quantified muscle degeneration and estimated muscle activity. In *C. elegans*, the number of body-wall muscle cells is fixed from one individual to the other (95 muscle cells). By contrast to mammals, *C. elegans* muscle cells are mononuclear and do not fuse. Moreover, as *C. elegans* lacks satellite cells and therefore, regenerative capabilities, each muscle cell can be individually followed *in vivo* throughout its whole life^59,60^, and muscle degeneration can be quantified by actin staining allowing to visualize and to count abnormal or absent muscle cells. Wild-type and *dys-1(cx18)* single mutant worms did not present abnormal muscle cells; nor did *drp-1(tm1108)* mutant worms or *eat-3* and *fzo-1* overexpressing worms (Figs 2A and S3A)^40^. *hlh-1(cc561ts)* mutant worms exhibited a weak number of abnormal muscle cells^40^. By contrast, *dys-1(cx18);hlh-1(cc561ts)* mutant worms exhibited a substantial number of abnormal muscle cells (Fig. 2A)^40^. Interestingly, lack of DRP-1 greatly reduced the number of abnormal muscle cells in *dys-1(cx18);hlh-1(cc561ts)* mutant worms (7,1 +/− 0,15 abnormal muscle cells by two quadrants in *dys-1(cx18);hlh-1(cc561ts)* mutant *vs* 3,3 +/− 0,20 in *dys-1(cx18);hlh-1(cc561ts);drp-1(tm1108)* mutant worms) (Fig. 2A) similarly to what was observed with *drp-1* RNAi (Fig. 2B)^45^. Both *eat-3* and *fzo-1* overexpression also reduced the number of abnormal muscle cells of *dys-1(cx18);hlh-1(cc561ts)* mutant worms (2,0 +/− 0,14 and 2,3 +/− 0,15 abnormal muscle cells by two quadrants in *dys-1(cx18);hlh-1(cc561ts);Ex[eat-3]* and in *dys-1(cx18);hlh-1(cc561ts);Ex[fzo-1]* mutant respectively *vs* 7,1 +/− 0,15 in *dys-1(cx18);hlh-1(cc561ts)* mutant worms) (Fig. 2A). These data were confirmed using two independent lines overexpressing *eat-3* and *fzo-1* (Fig. S3A). Conversely, we tested the effects of decreased mitochondrial fusion on muscle cells integrity of dystrophic worms. *fzo-1* or *eat-3* RNAi treatments had no effect on the actin staining of wild-type animals (data not shown). However, *dys-1(cx18);hlh-1(cc561ts)* mutant worms fed with either *fzo-1* or *eat-3* RNAi exhibited enhanced levels of muscle degeneration (number of abnormal muscle cells in *dys-1(cx18);hlh-1(cc561ts)* mutant worms fed with *eat-3* RNAi: 11,7 +/− 0,59 or *fzo-1* RNAi: 10,0 +/− 0,70 *vs* fed with the empty vector L4440: 6,6 +/− 0,44) (Fig. 2B). Collectively, it clearly appears that interfering with the mitochondrial dynamic balance can greatly impact dystrophin-dependent muscle degeneration in *C. elegan*s. Then, we wondered if the protective effects of mitochondrial dynamics on muscle degeneration could lead to positive effects on muscle fitness. To that extent, we quantified worm locomotion with thrashing assays and measured the number of worm bends during 30 seconds swimming sessions. Thrashing of *drp-1(tm1108)* mutant worms was slightly decreased compared to that of wild-type worms whereas *eat-3* and *fzo-1* overexpression or *eat-3* and *fzo-1* RNAi did not affect worm thrashing abilities (Fig. S3B,C). As expected, *dys-1(cx18);hlh-1(cc561ts)* mutant worms were unable to bend quickly and this poor muscle fitness was partially rescued by lowered DRP-1 levels (Fig. 2C,D). Moreover, *eat-3* and *fzo-1* overexpression also improved the muscle fitness of *dys-1(cx18);hlh-1(cc561ts)* mutant worms (Fig. 2C). These data were confirmed using three independent lines overexpressing *eat-3* and two independent lines overexpressing *fzo-1* (Fig. S3B). By contrast, *eat-3* or *fzo-1* RNAi could further decrease trashing abilities of *dys-1(cx18);hlh-1(cc561ts)* mutant worms (Fig. 2D). Together, our data indicate that decreasing mitochondrial fission or increasing mitochondrial fusion can induce a partial functional improvement of muscle cells in dystrophic worms whereas decreasing mitochondrial fusion could worsen muscle degeneration. In conclusion, the main molecular actors of mitochondrial dynamics, namely DRP-1, EAT-3 and FZO-1, appear to be implicated in the establishment of dystrophin-dependent muscle degeneration.

### Alterations of the mitochondrial network are early events of dystrophin-dependent muscle degeneration

We next wondered whether mitochondrial dynamics perturbations or apoptosis appears first in dystrophin-dependent muscle degeneration. We performed actin and TUNEL staining on *dys-1(cx18);hlh-1(cc561ts)* mutant or wild-type worms carrying the *ccIs4251* transgene. In wild-type worms, tubular and circular mitochondria were in equal proportions and organized along the actin network, which is arranged in filaments along the cell (Fig. 2E–H) and we could not detect any TUNEL positive cell (Fig. 2G). In *dys-1(cx18);hlh-1(cc561ts)* mutant worms, different stages of actin network perturbations were observed, likely reflecting various degrees of muscle degeneration. The actin staining can reveal an apparent totally preserved actin network or the apparition of wavelets or a more or less noticeable aggregation of actin. Muscle cells with mutated dystrophin and unperturbed actin network (Fig. 2I-I’) could be associated with either wild-type-like mitochondria (Fig. 2J) or mitochondria that appeared to be fragmented (Fig. 2J’) and we could not detect any TUNEL staining under these conditions (Fig. 2K-K’). It is noteworthy that weak actin perturbations (Fig. 2I”) were always associated with increased mitochondrial circularity in *dys-1(cx18);hlh-1(cc561ts)* mutant worms (Fig. 2J”) but without any signs of apoptosis (Fig. 2K”). Finally, strong actin perturbations due to mutated dystrophin (Fig. 2I”’) were concomitant with circular mitochondria (Fig. 2J”’) and TUNEL positive staining (Fig. 2K”’). Together, these observations suggest that mitochondrial dynamics dysfunction is an early event in the molecular mechanisms leading to progressive muscle degeneration. Changes in mitochondrial morphologies are then followed by actin network perturbations and will eventually lead to apoptosis.

### Cleavage of DRP-1 by CED-3 is required for dystrophin-dependent muscle degeneration but is dispensable for regulating mitochondrial fission

**Figure 3:**
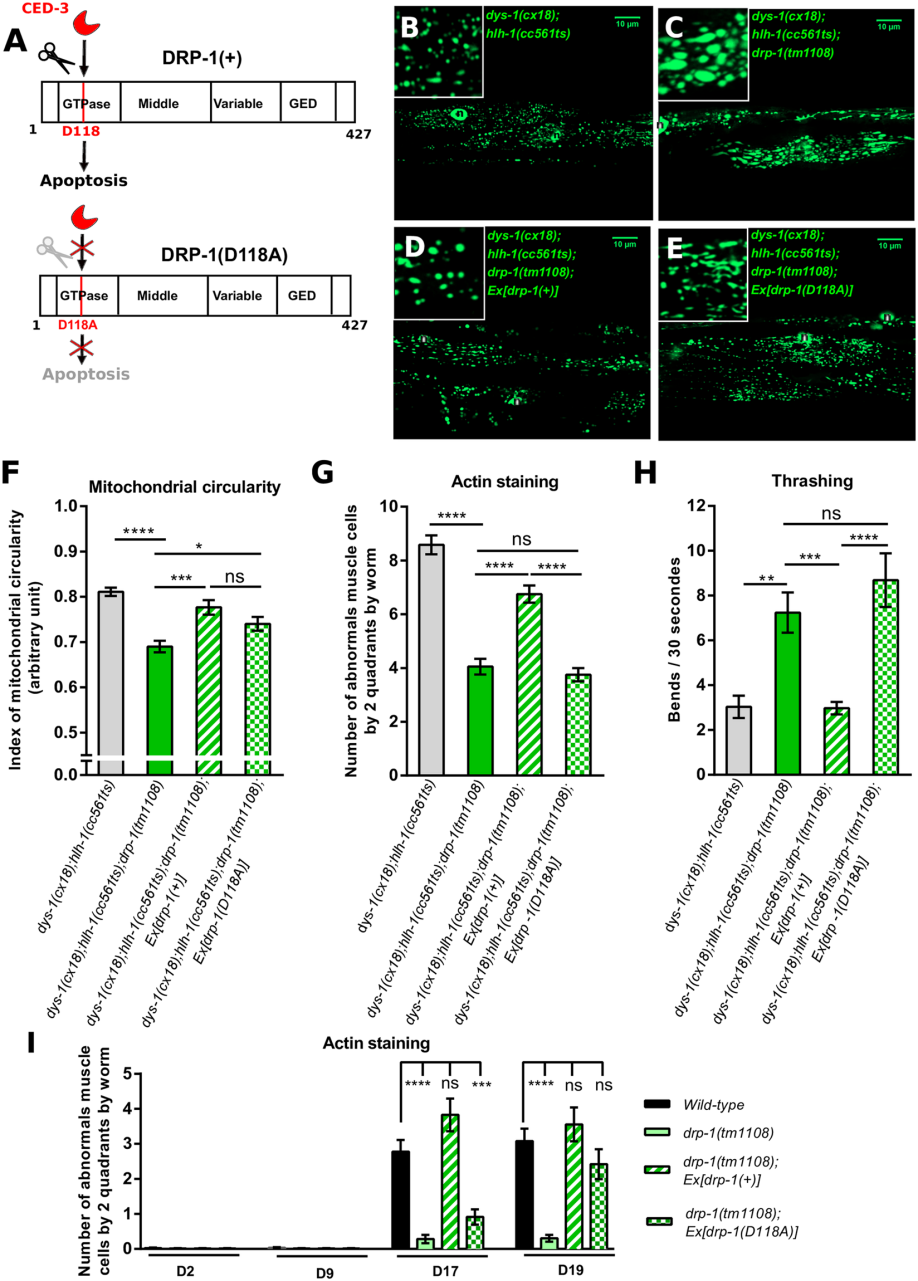
Cleavage of DRP-1 by CED-3 is required for dystrophin-dependent muscle degeneration but is dispensable for regulating mitochondrial fission. (**A**) Schematic representation of the wild-type DRP-1 construct (DRP-1(+)) and of the DRP-1 construct with CED-3 cleavage site mutated due to a change of amino acid D118A (DRP-1(D118A)). The wild-type DRP-1 construct DRP-1(+) is supposed to play a role in mitochondrial dynamics and in apoptosis, whereas the DRP-1 construct with D118A mutation DRP-1(D118A) is likely to be dispensable for apoptosis^31^. Representative confocal images of the mitochondrial network organization visualized with the *ccIs4251* transgene in *C. elegans* body wall muscle cells of: (**B**) *dys-1(cx18);hlh-1(cc561ts)* mutant worms; (**C**) *dys-1(cx18);hlh-1(cc561ts);drp-1(tm1108)* mutant worms; (**D**) *dys-1(cx18);hlh-1(cc561ts);drp-1(tm1108)* mutant worms expressing *drp-1*(+) and (**E**) *dys-1(cx18);hlh-1(cc561ts);drp-1(tm1108)* mutant worms expressing *drp-1(D118A)*. Scale bar 10 μm. (x630). n indicates nuclei. (**F**) Quantification of mitochondrial circularity in each of the indicated strains (n = 30 worms at least). (**G**) Number of abnormal *C. elegans* body wall muscle cells by two quadrants quantified by phalloïdin staining in each of the indicated strains (n = 60 worms). (**H**) Quantification of worm trashing in each of the indicated strains (n = 45 worms at least). All the experiments were performed on L4 + 3 day-old worms. One-way ANOVA, Tukey’s multiple comparisons test. (**I**) Number of abnormal *C. elegans* body wall muscle cells by two quadrants quantified by phalloïdin staining in wild-type worms; *drp-1(tm1108)* mutant worms; *drp-1(tm1108)* mutant worms expressing *drp-1*(+); *drp-1(tm1108)* mutant worms expressing *drp-1(D118A)* at day 2, 9, 17 and 19 of adulthood of the nematode (n = 63 worms at least). Student test. Data represent the mean obtained by pooling at least three independent assays. Errors bars represent SEM *p < 0.05 **p < 0.01 ***p < 0.001 ****p < 0,0001 ns. indicates that the mean is not statistically significantly different from the mean obtained in control worms.

The links between mitochondrial dynamics and cell death have been extensively studied^16,31,56,61,62^. In *C. elegans*, the importance and the exact roles of DRP-1 in developmental apoptosis are still controversial^31,56^. A fraction of DRP-1 can be cleaved *in vitro* by the caspase CED-3, leading to the formation of DRP-1 complexes containing both, full-length DRP-1 and DRP-1^119–712^, which are suspected to play a role in apoptosis during worm development. This cleavage by CED-3 may redirect DRP-1 activity towards apoptotic mitochondrial elimination in cell corpses^31^. Importantly, CED-3 cleavage of DRP-1 is unlikely to be implicated in mitochondrial fission^31^ suggesting that the roles of DRP-1 in mitochondrial dynamics and cell death might be distinguished. We wondered whether the role of DRP-1 in cell death is required for muscle degeneration in a dystrophin-deficient background. To this end, we used two genetic constructions of DRP-1: (*i*) a wild-type *drp-1* transgene called *drp-1*(+) and (*ii)* a transgene of *drp-1* with the CED-3 cleavage site mutated called *drp-1(D118A)* (Fig. 3A). The presence of this mutation prevents the cleavage of DRP-1 by CED-3 rendering DRP-1 presumably unable to act in cell death^31^. We compared the indexes of mitochondrial circularity of *dys-1(cx18);hlh-1(cc561ts)* and *dys-1(cx18);hlh-1(cc561ts);drp-1(tm1108)* mutant worms expressing, or not, *drp-1*(+) or *drp-1(D118A)* transgenes. As shown above, *dys-1(cx18);hlh-1(cc561ts)* mutant worms presented fragmented mitochondria (Figs 1F and 3B) with a high index of mitochondrial circularity that can be lowered in absence of DRP-1 (Figs 1G,J and 3C,F). *dys-1(cx18);hlh-1(cc561ts);drp-1(tm1108)* mutant worms expressing DRP-1(+) or DRP-1(D118A) showed important mitochondrial fragmentation, loss of the mitochondria bubbles caused by the *drp-1(tm1108)* mutation^7^ and an increased index of mitochondrial circularity compared to that of *dys-1(cx18);hlh-1(cc561ts);drp-1(tm1108)* mutant worms (Fig. 3D–F). These results demonstrate that lack of DRP-1 is responsible for the particular mitochondrial pattern exhibited by *dys-1(cx18);hlh-1(cc561ts);drp-1(tm1108)* mutant worms and that both *drp-1*(+) and *drp-1(D118A)* transgene are able to induce mitochondrial fission. Our data confirmed that cleavage of DRP-1 by CED-3 is at least in part dispensable for its function in regulating mitochondrial fission.

We next tested the effects of expressing *drp-1*(+) or *drp-1(D118A)* transgenes on muscle degeneration and fitness of *dys-1(cx18);hlh-1(cc561ts);drp-1(tm1108)* mutant worms. As shown above, *dys-1(cx18);hlh-1(cc561ts)* mutant worms exhibited an important number of abnormal muscle cells and weak thrashing abilities (Figs 2A,C and 3G,H) and both phenotypes were attenuated by the absence of DRP-1 (Figs 2A,C and 3G,H). The introduction of *drp-1*(+) transgene into *dys-1(cx18);hlh-1(cc561ts);drp-1(tm1108)* mutant worms provoked an increased number of abnormal muscle cells and a drastic thrashing decrease (6,75 +/− 0,32 abnormal muscle cells and 2,9+/−0,28 bends/30 seconds in *dys-1(cx18);hlh-1(cc561ts);drp-1(tm1108);Ex[drp-1*(+)*]* mutant *vs* 8,59 +/− 0,35 abnormal muscle cells and 7,4 +/− 0,90 bends/30 seconds in *dys-1(cx18);hlh-1(cc561ts);drp-1(tm1108)* mutant worms) (Fig. 3G,H). This demonstrates that DRP-1 takes active part in both the muscle cells integrity and movement defects phenotypes of *dys-1(cx18);hlh-1(cc561ts)* mutant worms. We hypothesized that if *drp-1(D118A)* construct is unable to increase hallmarks of muscle degeneration in the *dys-1(cx18);hlh-1(cc561ts);drp-1(tm1108)* mutant worms that would indicate that the role of DRP-1 in cell death is required for dystrophin-dependent muscle degeneration. We found that introducing *drp-1(D118A)* construct into *dys-1(cx18);hlh-1(cc561ts);drp-1(tm1108)* mutant worms affected neither the number of abnormal muscle cells nor thrashing (Fig. 3G,H). Similar results were obtained with at least two independent lines expressing DRP-1(D118A) (Fig. S4A,B). The mutated form of DRP-1 is unable to counteract the positive effects of the absence of DRP-1 on the muscle degeneration of *dys-1(cx18);hlh-1(cc561ts)* mutant worms. This strongly suggests that caspase cleavage of DRP-1 is required for dystrophin-dependent muscle degeneration in *C. elegans*.

### Cleavage of DRP-1 by CED-3 is required for age-dependent muscle degeneration

Physiological processes such as aging can also induce a type of muscle degeneration called sarcopenia. Sarcopenia, which results in the gradual loss of skeletal muscle mass in the elderly, is a major factor determining the decline in overall health and loss of autonomy of the aging population. In wild-type *C. elegans*, we revealed the spontaneous apparition of more than two abnormal muscle cells by muscle quadrant at days 17 and 19 of adulthood (Fig. 3I) accompanied by a gradual loss of locomotion as the animal ages (Fig. S5). The *drp-1(tm1108)* mutant worms showed impaired mobility early in adulthood life, which could be explained by energy production impairment^63,64^. Importantly, in absence of DRP-1, the apparition of abnormal muscle cells, over aging, was a very rare event (Fig. 3I). Yet, locomotion defects due to age cannot be improved by the absence of DRP-1. Our data demonstrate that DRP-1 is involved in both dystrophin-dependent and age-dependent muscle degeneration but is unlikely to be the key factor for locomotion defects that are observed over aging. To understand if the apoptotic functions of DRP-1 are important for age-dependent muscle degeneration as it is for dystrophin-dependent muscle degeneration, we analyzed *drp-1(tm1108)* mutant worms expressing either the wild-type *drp-1* transgene (*drp-1*(+)) or the *drp-1* transgene with the CED-3 cleavage site mutated (*drp-1(D118A))* supposed to be inefficient in apoptosis (Fig. 3A). At day 17 and 19 of adulthood, *drp-1(tm1108)* mutant worms with *drp-1*(+) transgene exhibited apparition of abnormal muscle cells to the same extent as wild-type worms, demonstrating a role for DRP-1 in age-related muscle degeneration. At day 17 of adulthood, *drp-1(tm1108)* mutant worms expressing the *drp-1(D118A)* transgene presented very few abnormal muscle cells (Fig. 3I). By contrast, at day 19 of adulthood, *drp-1(tm1108)* mutant worms expressing the *drp-1(D118A)* transgene exhibited nearly the same level of muscle degeneration than wild-type worms (Fig. 3I). Collectively, our data suggest a role for DRP-1 through apoptotic-like processes in both dystrophin-dependent and age-related muscle degeneration.

### The canonical apoptosis pathway mediates dystrophin-dependent muscle degeneration

**Figure 4:**
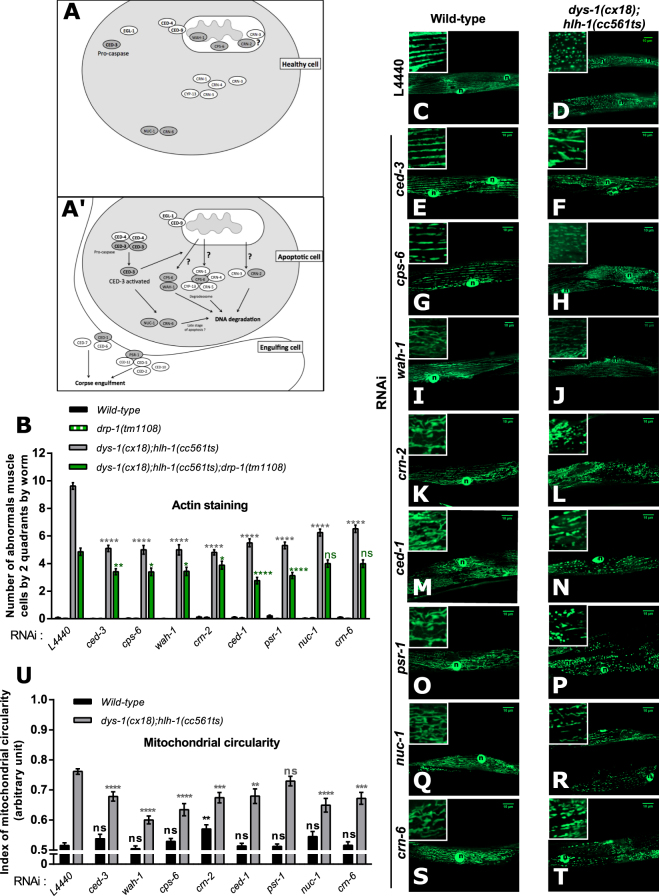
Genetic manipulation of apoptosis impacts muscle degeneration and mitochondrial dynamics of *dys-1(cx18);hlh-1(cc561ts)* mutant worms. Simplified schematic of the sub-cellular localization, in healthy (**A**) and apoptotic (**A’**) cells, of the main apoptotic factors involved in the inducing and execution phases of apoptosis in *C. elegans*. Gene names in dark gray indicate the genes, which knock-downs by RNAi, were tested on different phenotypes of *dys-1(cx18);hlh-1(cc561ts)* mutant and wild-type worms. (**B**) Number of abnormal *C. elegans* body wall muscle cells by two quadrants quantified by phalloïdin staining in wild-type worms or *drp-1(tm1108)* mutant worms or *dys-1(cx18);hlh-1(cc561ts)* mutant worms or *dys-1(cx18);hlh-1(cc561ts);drp-1(tm1108)* mutant worms fed either with the empty vector L4440 or with the indicated RNAi against executors of cell death (n = 90 worms at least). Representative confocal images of the mitochondrial network organization visualized with the *ccIs4251* transgene in *C. elegans* body wall muscle cells of: (**C**) wild-type worms or (**D**) *dys-1(cx18);hlh-1(cc561ts)* mutant worms fed with empty vector L4440; of (**E**) wild-type worms or (**F**) *dys-1(cx18);hlh-1(cc561ts)* mutant worms fed with *ced-3*RNAi; of (**G**) wild-type worms or (**H**) *dys-1(cx18);hlh-1(cc561ts)* mutant worms fed with *cps-6* RNAi; of (**I**) wild-type worms or (**J**) *dys-1(cx18);hlh-1(cc561ts)* mutant worms fed with *wah-1* RNAi; of (**K**) wild-type worms or (**L**) *dys-1(cx18);hlh-1(cc561ts)* mutant worms fed with *crn-2* RNAi; of (**M**) wild-type worms or (**N**) *dys-1(cx18);hlh-1(cc561ts)* mutant worms fed with *ced-1* RNAi; of (**O**) wild-type worms or (**P**) *dys-1(cx18);hlh-1(cc561ts)* mutant worms fed with *psr-1* RNAi; of (**Q**) wild-type worms or (**R**) *dys-1(cx18);hlh-1(cc561ts)* mutant worms fed with *nuc-1* RNAi and of (**S**) wild-type worms or (**T**) *dys-1(cx18);hlh-1(cc561ts)* mutant worms fed with *crn-6* RNAi. Scale bar 10 μm. (x630). n indicates nuclei. (**U**) Quantification of mitochondrial circularity in each of the indicated conditions (n = 27 worms at least). Note that quantitative PCR data showed that *nuc-1* and *crn-6* mRNA expression were reduced, in all strains, by ~50% and 70% upon *nuc-1* and *crn-6* RNAi treatments, respectively (Fig. S6). All the experiments were performed on L4 + 3 day-old worms. One-way ANOVA, Dunnett’s multiple comparisons test. Data represent the mean obtained by pooling at least three independent assays. Errors bars represent SEM **p < 0.01 ***p < 0.001 ****p < 0.0001 ns. indicates that the mean is not statistically significantly different from the mean obtained in control conditions.

Having established that the functions of DRP-1 in apoptosis play a key role in dystrophin-dependent muscle degeneration, we asked whether other executors of apoptosis could be important. For this, we performed RNAi experiments against several executors of cell death and tested the effects of each RNAi constructs on mitochondrial morphology and the number of abnormal cells in *dys-1(cx18);hlh-1(cc561ts)* mutant worms. We thus down-regulated CED-3, required for the execution of apoptosis *via* its activation by CED-4 (APAF-1 homolog in mammals) and for DRP-1 to act in apoptosis (Fig. 4A-A’)^65,66^. We have also tested the effects of RNAi-mediated down-regulation of five actors implicated in the three sub-pathways involved in DNA degradation. First, WAH-1 (AIF homolog in mammals) and CPS-6 (EndoG homolog in mammals) which, upon cell death signal, are released from mitochondria and can interact together to translocate to the nucleus where they participate to DNA degradation^67^. CPS-6 can also interact with CRN-1, CRN-4, CRN-5 and CYP-13 to form the degradeosome^68^. Second, CRN-2 likely to encode an homolog of the TatD nuclease (TATDN1 homolog in mammals) and predicted to be mitochondrial^68^. CRN-2 acts in the same pathway as CRN-3^68^. Third, NUC-1 and CRN-6 (two DNase II homologs in mammals) that seem involved in the degradation of DNA debris from apoptotic cells in late stage of apoptosis^68,69^. Concerning corpse engulfment, important for the phagocytic process^70^, we have tested the effects of knocking-down CED-1, which is involved in cell-corpse recognition with CED-6 and CED-7^71^ and PSR-1, which participates in the migration of engulfing cell^72,73^.

First, we performed actin staining and counted the number of abnormal muscles cells in *dys-1(cx18);hlh-1(cc561ts)* mutant worms fed with bacteria expressing either the empty vector L4440 or RNAi constructs against *ced-3* or each of the above described cell death executors tested. *ced-3* RNAi treatment dramatically decreased the number of abnormal muscle cells compared to that observed in *dys-1(cx18);hlh-1(cc561ts)* mutant worms fed with the empty vector L4440 (5,1 +/− 0,22 abnormal muscle cells for *dys-1(cx18);hlh-1(cc561ts)* fed with *ced-3* RNAi *vs* 9,63 +/− 0,24 abnormal muscle cells *for dys-1(cx18);hlh-1(cc561ts)* fed with L4440) (Fig. 4B) confirming that CED-3 plays an important role in dystrophin-dependent muscle degeneration. Interestingly, degenerin-dependent muscle degeneration in *C. elegans* also depends on CED-3^74^. All the RNAi against executors of cell death tested provoked a decrease in the number of abnormal muscle in *dys-1(cx18);hlh-1(cc561ts)* mutant worms (*dys-1(cx18);hlh-1(cc561ts)* mutant worms fed with: *cps-6* RNAi: 5,01 +/− 0,32 abnormal muscle cells; *wah-1* RNAi: 5,00 +/− 0,37 abnormal muscle cells; *crn-2* RNAi: 4,81+/0,19 abnormal muscle cells; *nuc-1* RNAi: 6,25 +/− 0,25 abnormal muscle cells; *crn-6* RNAi: 6,53 +/− 0,26 abnormal muscle cells; *ced-1* RNAi: 5,50 +/− 0,29 abnormal muscle cells; *psr-1* RNAi: 5,31 +/− 0,24 abnormal muscle cells *vs dys-1(cx18);hlh-1(cc561ts)* mutant worms fed with the empty vector L4440: 9,63 +/− 0,24 abnormal muscle cells) (Fig. 4B). Our data indicates that the canonical apoptotic pathway, which has been extensively described in developmental apoptosis^75^, also participates in cell death upon dystrophin-muscle degeneration.

Then, we wondered whether DRP-1 was required for CED-3 and others cell death executors to impact dystrophin-dependent muscle degeneration. For that, we tested the effects of the different RNAi presented above on muscle degeneration of *dys-1(cx18);hlh-1(cc561ts);drp-1(tm1108)* mutant worms. The effects of *ced-3* RNAi and of the *drp-1(tm1108)* mutation on muscle cells of *dys-1(cx18);hlh-1(cc561ts)* were cumulative indicating that CED-3 probably has an additional role in dystrophin-dependent muscle degeneration, which is DRP-1-independent. Moreover, knock-down of *ced-1* and *psr*-*1*, involved in corpse engulfment, could also further decrease the muscle degeneration of *dys-1(cx18);hlh-1(cc561ts);drp-1(tm1108)* mutant worms (*dys-1(cx18);hlh-1(cc561ts)* mutant worms fed with: *ced-1* RNAi: 2,77 +/− 0,24 abnormal muscle cells; *psr-1* RNAi: 3,13 +/− 0,22 abnormal muscle cells *vs dys-1(cx18);hlh-1(cc561ts)* mutant worms fed with the empty vector L4440: 4,86 +/− 0,26 abnormal muscle cells) (Fig. 4B). Similarly, knock-down of three genes that are involved in DNA degradation (*cps-6*, *wah-1* and *crn-2)* could further decrease muscle degeneration of *dys-1(cx18);hlh-1(cc561ts);drp-1(tm1108)* mutant worms fed with the empty vector L4440 (*dys-1(cx18);hlh-1(cc561ts)* mutant worms fed with: *cps-6* RNAi: 2,33 +/− 0,29 abnormal muscle cells; *wah-1* RNAi: 3,68 +/− 0,33 abnormal muscle cells; *crn-2* RNAi: 3,88 +/− 0,29 abnormal muscle cells *vs dys-1(cx18);hlh-1(cc561ts)* mutant worms fed with L4440: 4,86 +/− 0,26 abnormal muscle cells) (Fig. 4B). By contrast, NUC-1 and CRN-6, final actors of DNA degradation during the apoptotic process, appeared to act in the same pathway and downstream of DRP-1 as their inactivation had no significant effects on muscle degeneration of *dys-1(cx18);hlh-1(cc561ts);drp-1(tm1108)* mutant worms although *nuc-1* and *crn-6* RNAi decreased muscle degeneration in *dys-1(cx18);hlh-1(cc561ts)* mutant worms (Fig. 4B). None of the tested RNAi treatments had an effect on the muscle degeneration of wild-type or *drp-1(tm1108)* mutant worms (Fig. 4B). Based on these data, we propose two hypothesis: (*i*) DRP-1 may act in parallel of the DNA degradation pathway in dystrophin-dependent muscle cell death, (*ii*) DRP-1 may act upstream of the DNA degradation pathways.

We then tested the effect of cell death executors on mitochondrial dynamics. None of the RNAi tested (except for *crn-2* RNAi) had an effect on mitochondrial morphologies in wild-type worms carrying the *ccIs4251* transgene (Fig. 4C–U). In a *dys-1(cx18);hlh-1(cc561ts)* mutant background, all the tested RNAi, with exception of *psr-1* RNAi (Fig. 4O,P), diminished mitochondrial fragmentation and circularity indexes when compared to mutant worms fed with empty vector (index of mitochondrial circularity in *dys-1(cx18);hlh-1(cc561ts)* mutant worms fed with: *wah-1* RNAi: 0,60 +/− 0,013; *cps-6* RNAi: 0,63 +/− 0,020; *crn-2* RNAi: 0,67 +/− 0,017; *nuc-1* RNAi: 0,64 +/− 0,022; *crn-6* RNAi: 0,67 +/− 0,019; *ced-1* RNAi: 0,68 +/− 0,024 *vs dys-1(cx18);hlh-1(cc561ts)* mutant worms fed with the empty vector L4440: 0,76 +/− 0,009) (Fig. 4C–U). Thus, decreasing the expression of apoptotic factors can partially rescue mitochondrial circularity of *dys-1(cx18);hlh-1(cc561ts)* mutant worms.

## Discussion

Apoptotic muscle fibers have been frequently associated with Duchenne Muscular Dystrophy^76–79^ and sarcopenia^80–83^ in various species including Human. However, the exact molecular mechanisms leading to muscle apoptosis upon dystrophin deficiency or aging remain unclear. Here, we revealed a DRP-1-dependent apoptotic mechanism that participates in dystrophin-dependent and age-dependent muscle degeneration. DRP-1 is the principal and universal actor of mitochondrial fission. In mammals, mitochondrial fragmentation induced by DRP-1 can trigger apoptosis^84^. In *C. elegans*, a previous study suggested a marginal role for DRP-1 in apoptosis during development using the mutation *drp-1*(*tm1108*)^31^, whereas another study showed that the dominantly interfering mutation *drp-1*(*K40A*) caused mitochondria to form large blebs and induced excessive apoptosis in embryos^56^. Our study reveals that DRP-1 can play a key role in apoptosis that takes place during cellular stresses provoked either by the loss of dystrophin function or by aging. Our data also demonstrate that EAT-3 and FZO-1 overexpression in dystrophin deficient worms can reduce both mitochondrial fragmentation and muscle degeneration. A recent study showed that in mice, muscle-specific deletion of OPA1 (EAT-3 homolog in *C. elegans*) induces a precocious aging phenotype with muscle loss and weakness^85^ and a reduced muscle mass has been also reported in mice ablated for both *Mfn1* (FZO-1 homolog in *C. elegans*) and *Mfn2*^85^. These observations are nicely corroborating our findings and the fact that levels of mitochondrial fusion greatly impact muscle degeneration. Our data also suggest that manipulating mitochondrial fusion is sufficient to decrease apoptosis in dystrophic worms. In *C. elegans*, the direct links between mitochondrial dynamics and developmental apoptosis are highly controversial^25,26^; our study suggests that, upon cellular stress such as dystrophin deficiency, the major actors of mitochondrial dynamics in worms, namely DRP-1, EAT-3 and FZO-1, can directly interplay with the apoptotic pathways.

We also showed that DRP-1 cleavage by the pro-caspase CED-3 is required for dystrophin-dependent and age-dependent muscle degeneration. Hence, our study demonstrates that DRP-1 sits downstream of CED-3 and strongly suggests that DRP-1 acts mostly *via* apoptosis rather than through mitochondrial fission to induce muscle cell death. In mammals, DRP-1-induced outer membrane fragmentation allows for release of mitochondrial pro-apoptotic factors such as cytochrome *c*, EndoG (CPS-6 in worms) or AIF (WAH-1 in worms)^13–15,86^. In *C. elegans* muscle cells, cytochrome *c* could be released from mitochondria to the cytosol during muscle degeneration^45,74^ but its role in activating APAF-1 (CED-4 homolog in worms) to form the apoptosome is unlikely to be conserved^87,88^. Strong evidence suggests that both WAH-1 (AIF homolog in mammals) and CSP-6 (EndoG homolog in mammals) can be released from the mitochondria to the nucleus to degrade DNA upon apoptotic stimuli in *C. elegans*^67,89^. Our data emphasize a function for WAH-1/CSP-6 in dystrophin-dependent muscle degeneration potentially downstream of DRP-1. One possibility is that DRP-1, once activated by CED-3 induces mitochondrial outer membrane permeabilization that will, in turn, allows for WAH-1/CSP-6 release from the mitochondria and translocation into the nucleus. However, we cannot exclude that DRP-1 acts in a parallel pathway of WAH-1/CSP-6. Our data strongly suggest that DRP-1 does not participate in corpse engulfment during apoptosis and is likely to act upstream of NUC-1 and CRN-6. Collectively, our data converge toward a mechanism that underlies DRP-1-dependent mitochondrial pathways activated downstream of CED-3 to impact dystrophin-dependent muscle degeneration. The cleavage of DRP-1 by caspase CED-3 seems to be restricted to *C. elegans*^31^ suggesting that it could be an ancestral role of CED-3 that was lost over evolution. However, we also found that diminution of CED-3 expression can decrease muscle degeneration of a dystrophin *C. elegans* mutant in a DRP-1-independent manner. Our data are consistent with the fact that maintenance of *C. elegans* muscle integrity during aging depends on CED-3^90^. Interestingly, skeletal muscles of aged rats exhibit a high level of activated caspase-3^91^ and muscles of Duchenne Muscular Dystrophy patients present an increase of both caspase-3 expression^92^ and activity^93^. Collectively, the role of the caspase-3 in muscle degeneration appears to be likely conserved among species.

Having established that DRP-1 acts mostly in apoptosis to impact muscle degeneration upon dystrophin deficiency, it would be interesting to investigate whether others molecular mechanisms than cleavage by caspase-3 can regulate DRP-1 activity^94^. Control of intracellular concentration of Ca^2+^ ([Ca^2+^]_i_) homeostasis is critical for the maintenance of muscle contraction and relaxation. Elevated [Ca^2+^]_i_ increases production of reactive oxygen species (ROS)^95^, activates calpain^96^, impairs autophagy^97^, increases mitochondrial Ca^2+^ accumulation^96^ and induces cell death^98,99^. Intriguingly, a drastic increase in the [Ca^2+^]_i_ in myofibers and myotubes deficient in dystrophin was observed in several studies^100,101^. In *mdx* mice, abnormal elevation of [Ca^2+^]_i_ is due to SERCA (sarco(endo)plasmic reticulum Ca^2+^ ATPase) inhibition suggesting an importance of the ER-dependent regulation of Ca^2+^ in Duchenne Muscular Dystrophy^102,103^. Moreover, muscle degeneration of the *dys-1(cx18);hlh-1(cc561ts)* mutants worms is likely to be a calcium-dependent^104,105^. Calcium is also a well-known activator of DRP-1. For instance, ER-calcium release induces uptake of calcium by mitochondria and DRP-1 activation allowing for calcium-dependent mitochondrial fission^106^. Calcineurin, a calcium-dependent phosphatase, dephosphorylates DRP1 and promotes mitochondrial fragmentation and cell vulnerability to apoptosis^107,108^. In neurons of *C. elegans*, phosphorylation of DRP-1 by Ca/calmodulin-dependent kinase II (CaMKII) inhibits DRP-1 activity^109^. One possibility is that the loss of dystrophin function could induce changes in intracellular calcium that would activate DRP-1 and cell death.

In conclusion, our findings point to mitochondrial dynamics as an early signaling hub that controls cell death in dystrophin-dependent muscle degeneration. A better understanding of the role of DRP-1 in muscle degeneration could allow in the long term to identify new targets for treatments of muscular dystrophies or to improve muscle degeneration in the elderly.

## Materials and Methods

### Strains

Most of the strains used were obtained from the *Caenorhabditis Genetic Center* with the exception of the *drp-1(tm1108)* strain provided by the National Bioresource Project for the Nematode to Sylvia Lee’s lab (Cornell University-Ithaca, NY-USA). N2 was used as the wild-type (WT) strain. The *dys-1(cx18);hlh-1(cc561ts)*^40^*, dys-1(cx18);hlh-1(cc561ts);drp-1(tm1108*) and the *dys-1(cx18);hlh-1(cc561ts);ccIs4251* strains were constructed using standard genetic methods. The *drp-1(tm1108)* strain, which has no detectable DRP-1 protein expression, is likely to represent a strong loss-of-function or null mutant^31^. The integrated *ccIs4251* transgene contains *Pmyo-3::Ngfp-lacZ* (nuclear localization of GFP) and *Pmyo-3::Mtgfp* (mitochondrial localization of GFP)^55^.

All strains were maintained at 15 °C on standard NGM (Nematode Growth Medium) agar plates seeded with *Escherichia coli* strain OP50 or with HT115 bacteria for RNAi experiments.

### Generation of *drp-1, eat-3, fzo-1* constructs and transgenic lines

All constructs were verified by sequencing and restriction fragment length. Sequences of the primers used for PCR are available upon request.

#### *drp-1*(+) transgene under control of endogenous *drp-1* promoter

A first 4.3 Kbp genomic fragment containing the 950 bp sequence upstream and the 2.5 Kbp coding region, and the 1.1 Kbp sequence downstream of the *drp-1* coding region was amplified from genomic DNA by PCR. A second 3.6 Kbp genomic fragment containing the 650 bp sequence upstream, the 2.5 Kbp coding region, and the 490 bp sequence downstream of the *drp-1* coding region was amplified from the first 4.3 Kbp fragment. Gibson Assembly® Master Mix and *KpnI* enzyme was used to introduce this second fragment in pBSC vector.

#### *drp-1(D118A)* under control of endogenous *drp-1* promoter

A first 4.3 Kbp genomic fragment containing the 950 bp sequence upstream, the 2.5 Kbp coding region, and the 1.1 Kbp sequence downstream of the *drp-1* coding region was amplified from genomic DNA by PCR. Two fragments of 1050 pb and 2640 pb with overlapping end in *drp-1* containing a mutation (A > G) on the CED-3 cleavage site of DRP-1 were amplified by PCR from the first 4.3 Kbp fragment. A 3.6 Kpb fragment containing the 650 bp sequence upstream, the 2.5 Kbp coding region with a mutation on the CED-3 cleavage site of DRP-1, and the 490 bp sequence downstream of the *drp-1* coding region was amplified from the two fragments described above by Fusion-PCR. Gibson Assembly® Master Mix and KpnI enzyme was used to introduce this second fragment in pBSC vector.

#### *eat-3*(+) transgene under control of endogenous *eat-3* promoter

First, two genomic fragments of 2.9 Kbp and 3.3 Kpb containing the 701 bp sequence upstream, the 4.7 Kbp coding region, and the 474 bp sequence downstream of the *eat-3* coding region were amplified from genomic DNA by PCR. Secondly, two fragments of 2.3 Kpb and 2.8 Kpb with overlapping end in *eat-3* were amplified by PCR from the first two fragments described above. A 5.1 Kpb fragment containing the 182 bp sequence upstream, the 4.7 Kbp coding region, and the 216 bp sequence downstream of the *eat-3* coding region was amplified from the two fragments described above by Fusion-PCR.

#### *fzo-1*(+) transgene under control of endogenous *fzo-1* promoter

A first 3.8 Kbp genomic fragment containing the 163 bp sequence upstream, the 2.8 Kbp coding region, and the 762 bp sequence downstream of the *fzo-1* coding region was amplified from genomic DNA by PCR. A second 3.6 Kbp genomic fragment containing the 113 bp sequence upstream, the 2.8 Kbp coding region, and the 702 bp sequence downstream of the *fzo-1* coding region was amplified from the first fragment described above.

For generation of transgenic animals: 10 ng/μl of transgene (*drp-1*(+) or *drp-1(D118A)* or *eat-3*(+) or *fzo-1*(+)); 2,5 ng/μl of the co-injection marker pCFJ90 (*Pmyo-2::MCherry::unc-54utr*) and 116,5 ng/μl Bluescript plasmid filler DNA were microinjected into the gonads of adult wild-type, *dys-1(cx18);hlh-1(cc561ts);drp-1(tm1108)* or *dys-1(cx18);hlh-1(cc561ts)* mutant worms using standard methods^110^. F1 progeny were selected on the basis of MCherry fluorescence. Individual F2 animals were isolated to establish independent lines.

### RNA interference

RNAi bacteria clones from the commercial *C. elegans* RNAi collection (Ahringher laboratory-Gene Service Inc) were cultured overnight in LB medium supplemented with 100 mg/ml ampicillin and 12.5 mg/ml tetracycline (LBAT) at 37 C. The day after, 0.01 volume of this pre-culture was added in 1 volume of LBAT and incubated at 37 C. At 0.6–0.8 DO, bacteria were concentrated 5 times and then 300 μL of the bacteria culture was seeded on NGM plates containing ampicillin (100 ug/mL) and tetracycline (12,5 μg/mL). Plates were allowed to dry at room temperature for at least 3 days. Then, plates were stored at 4 C during 2 months utmost. The day before the experiment, 4 mM IPTG was added to induce bacteria overnight at room temperature. Gravid worms were allowed to lay eggs during 6 hours on NGM plates seeded with RNAi bacteria carrying specific dsRNA-expressing bacteria and progeny grew on RNAi plates until the day of experiment. Unless indicated differently, experiments were performed at the age of 3 days after the L4 larval stage (L4 + 3 days). As RNAi efficiency control, *dpy-13* RNAi was performed in parallel and resulted in nearly 100% dumpy. As RNAi-negative control, plates seeded with HT115 carrying the empty vector RNAi L4440 were used.

### Phalloidin staining

Phalloidin staining was carried out as described previously^111^. Synchronized adult (day 2, 9, 17 and 19 of adulthood) animals were fixed in 1 ml of PBS supplemented with 20 μl of 37% formaldehyde, extracted with acetone at −20 °C, and incubated in 3U of phalloidinAlexa Fluor® 488 (Molecular Probes) for 2 h. Imager Z1 microscope was used for microscopic observations. Only the two most visible quadrants of each animal were counted for the quantification of muscle degeneration.

For aging experiments, 1.3 μg/mL 5-FU (5-Fluorouracil, Sigma) was added when the worms reached the L4 stage.

### Thrashing assay

L4 worms were transferred to a plate during 3 days. In the case of transgenic lines, only the MCherry-fluorescent L4 worms (expressing the co-injection marker *Pmyo-2::mCherry::unc-54utr*) were transferred. Synchronized adult animals (day 2, 9, 17 and 21 of adulthood), were moved onto a fresh 2% agarose 12 wells-plates containing M9 bufferpre-equilibrated at a temperature of 15 °C. Thrashing frequency was measured during the swimming sessions in the liquid. Videos of animal movements were acquired using a Macro Zoom microscope. The videos were recorded for 30 seconds at 30 frames per second. The thrashing frequency of the worms, measured in body bends per 30 seconds, was quantified using the open-source *wrMTrck* plugin for *Fiji* software^112^ (http://www.phage.dk/plugins/wrmtrck.html). One body bend was defined as a change indirection of bending at the midbody. For aging experiments, 1.3 μg/mL 5-FU (5-Fluorouracil, Sigma) was added when the worms reached the L4 stage.

### Mitochondrial morphology analysis

Gravid adults with integrated *ccIs4251* transgene were allowed to lay eggs on NGM plates for 6 hours before being removed from the plate. L4 + 3 day-old progeny worms were immobilized in 3.3 mM levamisole on a 2% agarose pad for acquisition. Confocal images of the vulva area of lived paralyzed worms were taken on a Zeiss LSM 510 Meta or Zeiss LSM 800 using a 63X oil objective. The percentage of muscle cells exhibiting interconnecting filaments in between mitochondria blebs was calculated after observation, with the unaided human eye of the experimenter, of each muscle cells with an increase of brightness of the confocal images. To quantify mitochondrial circularity, *Fiji* software and a homemade macro were used to process and to analyze captured images. To fix threshold, the inverse Fourier transformation (Inverse FFT) was used in order to minimize the noise and to sharpen the contrast between mitochondria and the background. Nucleus of each image was removed by hand (with the unaided human eye). « Analyze particles » command was used to obtain values for circularity (calculated by 4πArea/perimeter²) for each mitochondrion in an image, ignoring mitochondria <5 square-pixels or on the edge of the image. Mitochondrial circularity at 0 refers to a straight line whereas mitochondrial circularity at 1 refers to a perfect circle.

In order to determine the accuracy and consistency of the circularity measurements obtained with *Fiji*, we have compared the circularity indexes of 96 confocal images measured by visual counting and the circularity indexes measured by the macro processed. No statistical differences were found between the two methods.

### TUNEL assay

Synchronized L4 + 3 day-old worms were fixed in 1 ml of PBS supplemented with 20 μl of 37% formaldehyde, extracted with acetone at −20 °C, and incubated in 50 μL of TUNEL solution (*In Situ* Cell Death Detection Kit, Fluorescein Roche) for 1.5 hours at 37 C. After incubation, worms were washed 3 times in PBST for 10 min. Then, phalloïdin staining with phalloïdin Alexa Fluor® 633 (Molecular Probes) was performed like previously described above. Worms were then mounted in Vectorshield mounting medium with DAPI (Vector Laboratories) and visualized using either a Zeiss Axiophot microscope or Zeiss LSM 710 confocal microscope. Only the two most visible quadrants of each animal were counted for the quantification of TUNEL positives muscle cells.

### Statistical analyses

One-way ANOVA was used to test statistical differences between independent groups within the same experiment. Statistical significance was tested with Tukey and Dunnett post tests. Two-tailed unpaired Student’s t-test was used to examine direct differences between two independent groups.

No data sets were generated or analyzed during the current study.

## Electronic supplementary material

Supplementary information
