# Author Correction: DRP-1-mediated apoptosis induces muscle degeneration in dystrophin mutants

**DOI:** 10.1038/s41598-020-68522-0

**Published:** 2020-07-10

**Authors:** Charlotte Scholtes, Stéphanie Bellemin, Edwige Martin, Maïté Carre-Pierrat, Bertrand Mollereau, Kathrin Gieseler, Ludivine Walter

**Affiliations:** 1 Laboratory of Biology and Modelling of the Cell, UMR5239 CNRS/Ecole Normale Supérieure de Lyon, UMS 3444 Biosciences Lyon Gerland, Universite de Lyon, 69007 Lyon, France; 2 NeuroMyoGene Institute (INMG), Universite Lyon 1, CNRS UMR 5310, INSERM U1217, 69008 Lyon, France; 3 Biology of Caenorhabditis Elegans Facility, Universite Lyon 1, UMS3421, 69008 Lyon, France

Correction to: *Scientific Reports* 10.1038/s41598-018-25727-8, published online 09 May 2018

This Article contains an error. In Figure 4, the insert for panel K was inadvertently duplicated from panel O. The correct Figure 4 appears below as Figure 1.

**Figure 1:**
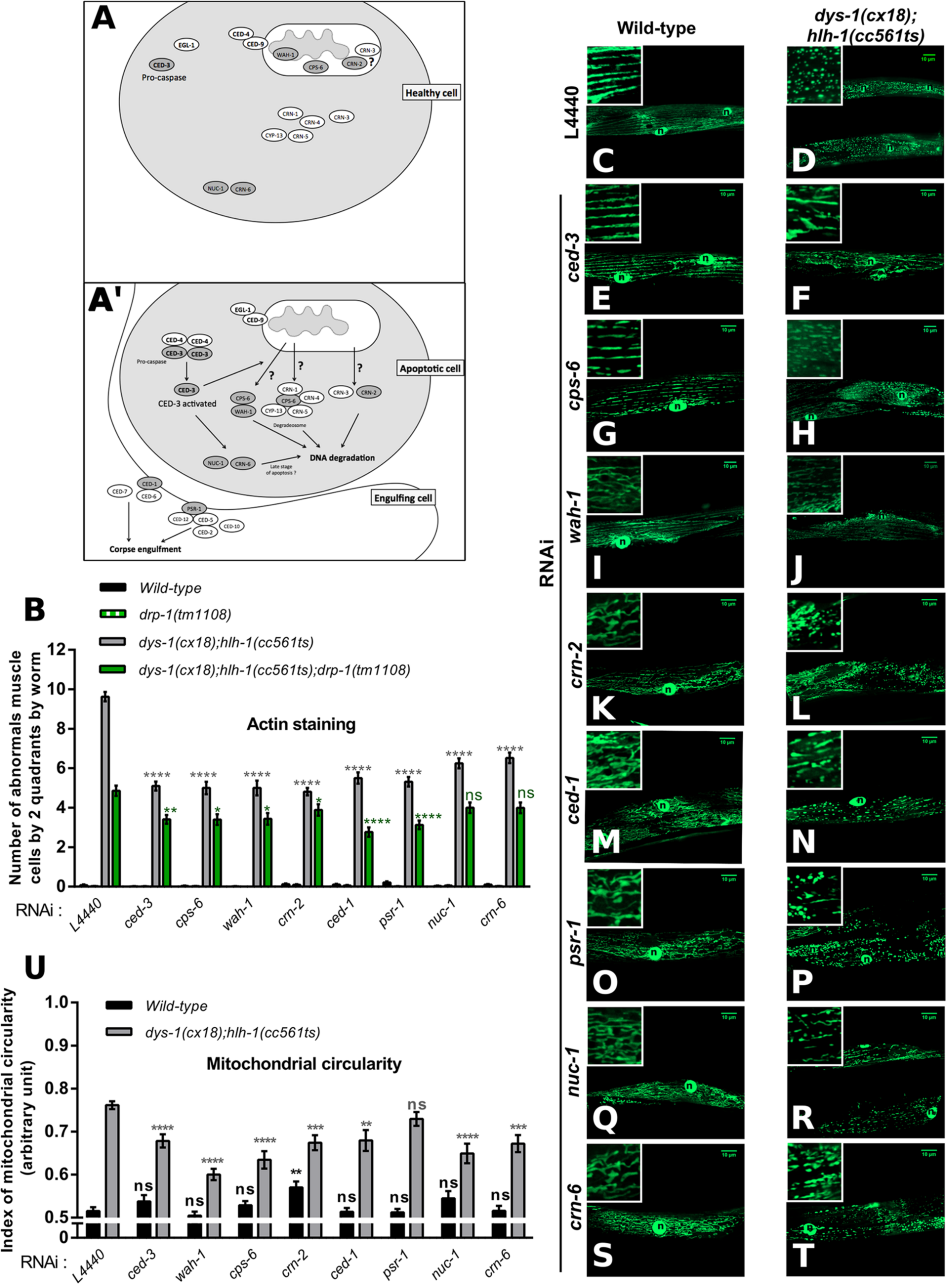
.

