# Supplementary material for: DRP-1-mediated apoptosis induces muscle degeneration in dystrophin mutants
Source: Sci Rep. 2018 May 9;8:7354. doi: 10.1038/s41598-018-25727-8 (PMC5943356; doi:10.1038/s41598-018-25727-8)
Supplement: Supplementary file 1 — Supplementary information [file 41598_2018_25727_MOESM1_ESM.docx]

Full title:

**DRP-1-mediated apoptosis induces muscle degeneration in dystrophin mutants**

Charlotte Scholtes^1^, Stéphanie Bellemin^2^, Edwige Martin^2^, Maïté Carre-Pierrat^3^, Bertrand Mollereau^1^, Kathrin Gieseler^2*^ and Ludivine Walter^1*^

^1^ Laboratory of Biology and Modelling of the Cell, UMR5239 CNRS / Ecole Normale Supérieure de Lyon, UMS 3444 Biosciences Lyon Gerland, Université de Lyon, Lyon 69007, France.

^2^ NeuroMyoGene Institute (INMG), Université Lyon 1, CNRS UMR 5310, INSERM U1217, Villeurbanne, France

^3^ Biology of *Caenorhabditis elegans* facility, Université Lyon 1, UMS3421, Villeurbanne, France

* Corresponding authors

E-mails: [ludivine.walter­@univ-lyon1.fr](mailto:ludivine.walter@univ-lyon1.fr) +33 4 72 72 87 90 (LW), [kathrin.gieseler@univ-lyon1.fr](mailto:kathrin.gieseler@univ-lyon1.fr) + 33 4 72 43 29 51 (KG)

# Supporting Information

##
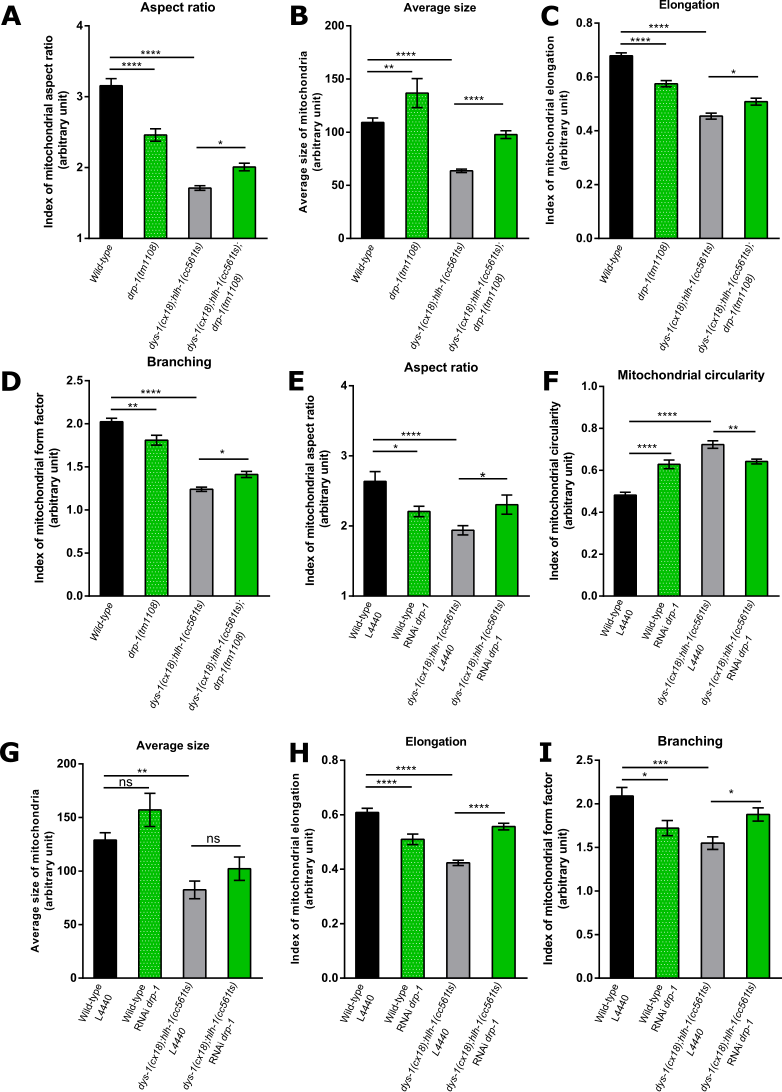


## S1 Fig. Effects of knocking-down *drp-1* on mitochondrial dynamics of wild-type and *dys-1(cx18);hlh-1(cc561ts)* mutant worms.

Quantification in wild-type, *drp-1(tm1108)* mutant, *dys-1(cx18);hlh-1(cc561ts)* mutant, and *dys-1(cx18);hlh-1(cc561ts);drp-1(tm1108)* mutant worms of: **(A)** mitochondrial aspect ratio (major axis/minor axis) ; **(B)** mitochondrial average size ; **(C)** mitochondrial elongation (1-(minor axis/major axis)) **(D)** mitochondrial branching (perimeter² / 4πArea). (n=60 worms at least). Quantification in wild-type and in *dys-1(cx18);hlh-1(cc561ts)* mutant worms fed either with empty vector L4440 or with *drp-1* RNAi of: **(E)** mitochondrial aspect ratio (major axis/minor axis) ; **(F)** mitochondrial circularity (4πArea/ perimeter²) ; **(G)** mitochondrial average size ; **(H)** mitochondrial elongation (1-(minor axis/major axis)) **(I)** mitochondrial branching (perimeter² / 4πArea). (n=30 worms at least). All the experiments were performed on L4 + 3 day-old worms. Data represent the mean. Errors bars represent SEM. One-way ANOVA, Tukey’s multiple comparisons test. * p < 0.05 ,** p < 0.01, *** p < 0.001, **** p < 0.0001. n.s. indicates that the mean is not statistically significantly different from the mean obtained in the control condition.


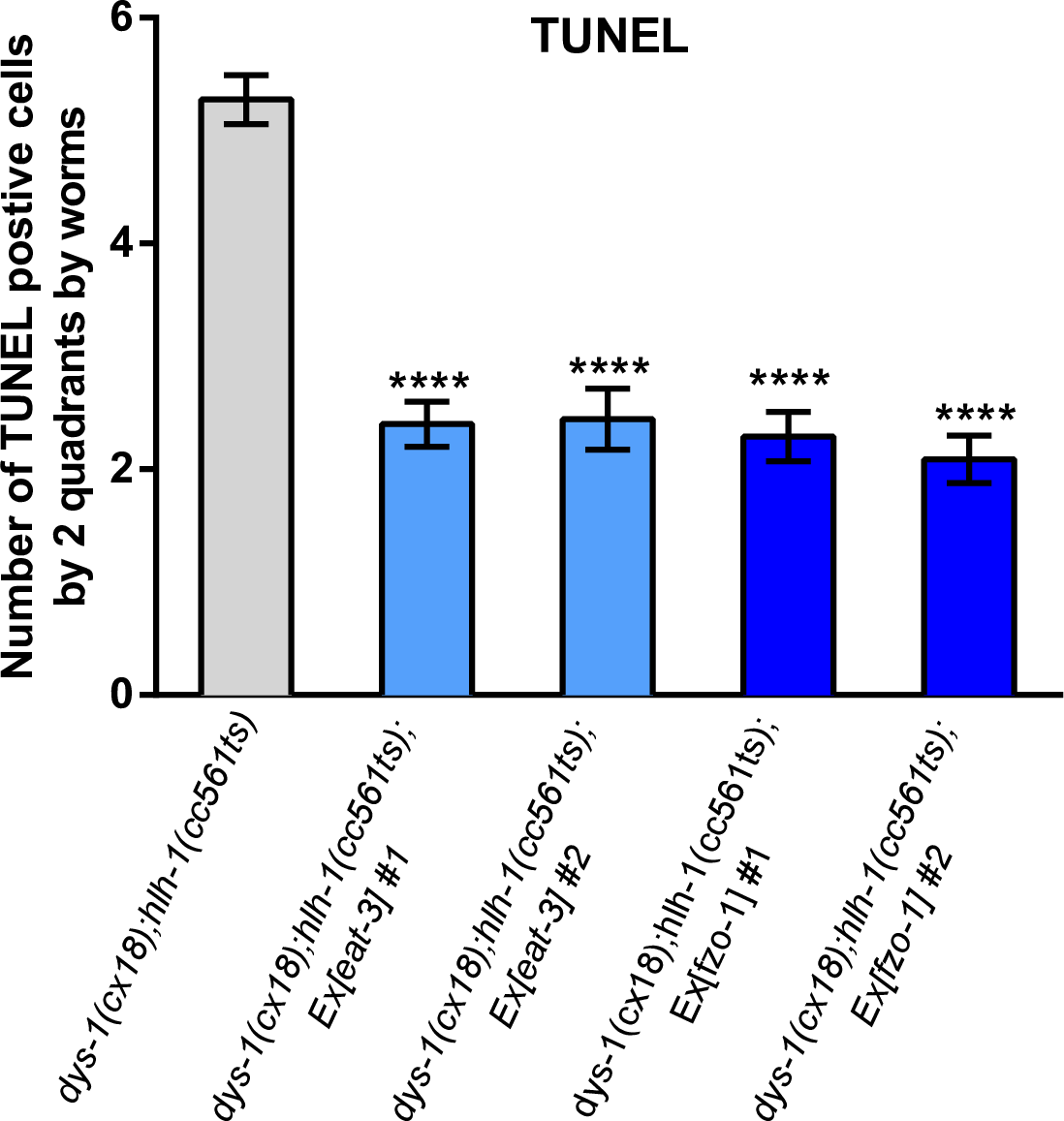


**S2 Fig. Overexpression of fusion genes decreases dystrophin-dependent apoptosis.**

Quantification of TUNEL positives muscle cells in *dys-1(cx18);hlh-1(cc561ts)* mutant worms with overexpression of *eat-3* or *fzo-1* (n=45 worms at least). # indicates independent generated lines. All the experiments were performed on L4 + 3 day-old worms. Data represent the mean obtained by pooling at least three independent assays. Errors bars represent SEM. One-way ANOVA, Tukey’s multiple comparisons test. * p < 0.05, ** p < 0.01, *** p < 0.001, **** p < 0.0001.

**
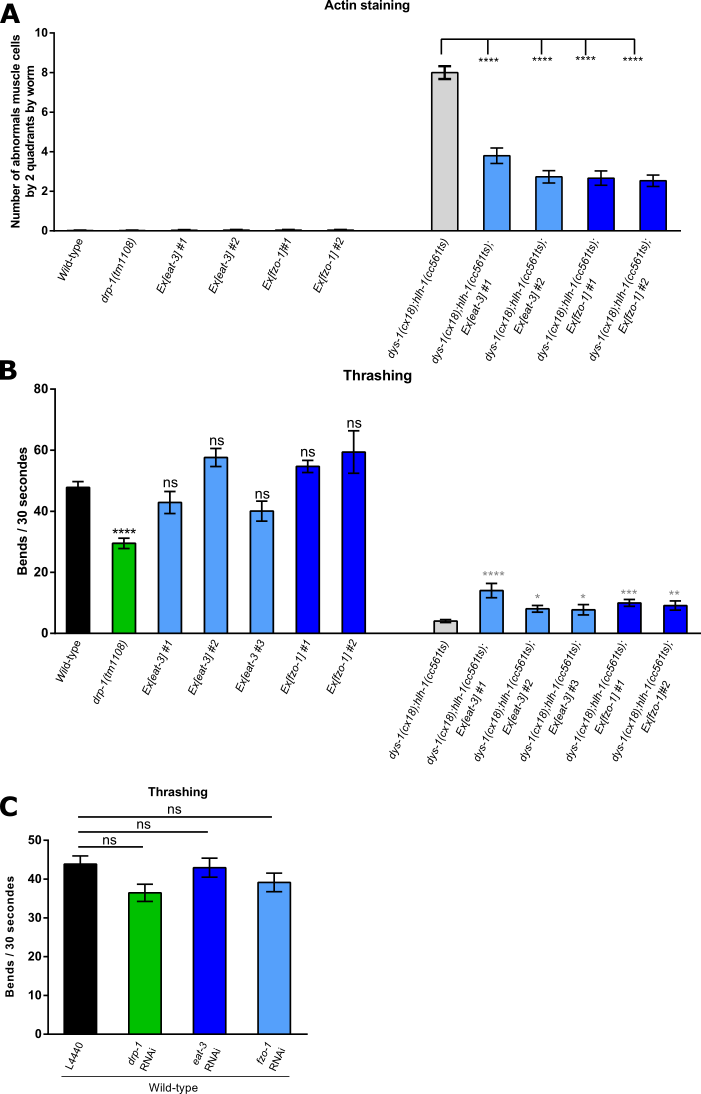
**

**S3 Fig. Effects of genetic manipulations of mitochondrial dynamics on dystrophin-dependent muscle degeneration and locomotion defects.**

**(A)** Number of abnormal *C. elegans* body wall muscle cells by two quadrants quantified by phalloïdin staining in each of the indicated strains (n=30 worms at least). **(B)** Quantification of worm trashing in each of the indicated strains (n=25 worms at least). # indicates independent generated lines. **(C)** Quantification of worm trashing in wild-type worms fed with the empty vector L4440 or with *drp-1* RNAi or *eat-3* RNAi or *fzo-1* RNAi (n=100 worms at least). All the experiments were performed on L4 + 3 day-old worms. Data represent the mean obtained by pooling at least three independent assays. Errors bars represent SEM. One-way ANOVA, Tukey’s multiple comparisons test. * p < 0.05, ** p < 0.01, **** p < 0.0001. n.s. indicates that the mean is not statistically significantly different from the mean obtained in the control condition.


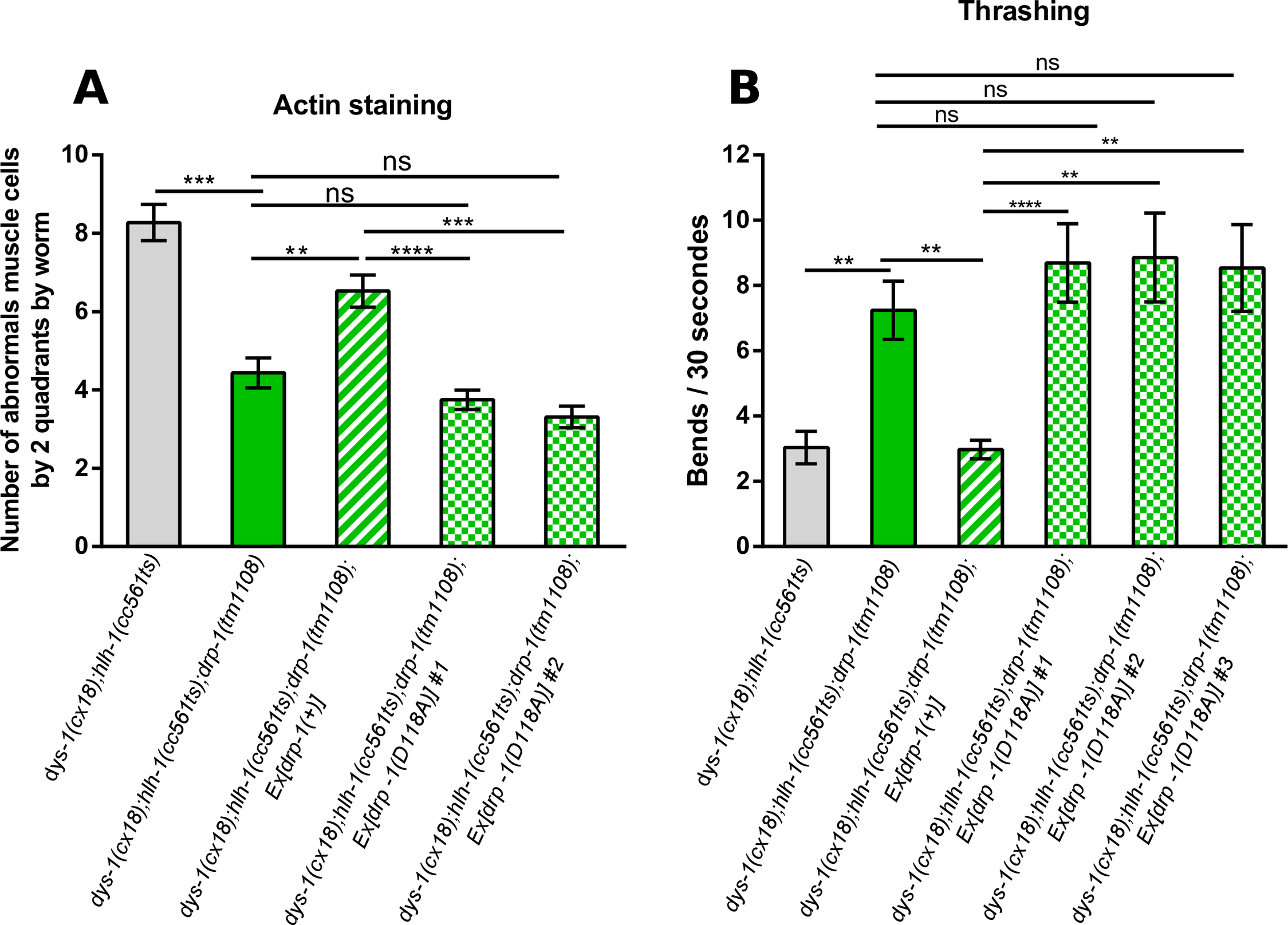


## S4 Fig. Cleavage of DRP-1 by CED-3 is required for dystrophin-dependent muscle degeneration but is dispensable for regulating mitochondrial fission.

**(A)** Number of abnormal *C. elegans* body wall muscle cells by two quadrants quantified by phalloidin staining in each of the indicated strains (n= 60 worms). **(B)** Quantification of worm trashing in each of the indicated strains (n=44 worms at least). # indicates independent generated lines. All the experiments were performed on L4 + 3 day-old worms. Data represent the mean obtained by pooling at least three independent assays. Errors bars represent SEM. One-way ANOVA, Tukey’s multiple comparisons test. ** p < 0.01, *** p < 0.001, **** p < 0.0001, n.s. indicates that the mean is not statistically significantly different from the mean obtained in the control condition.

**
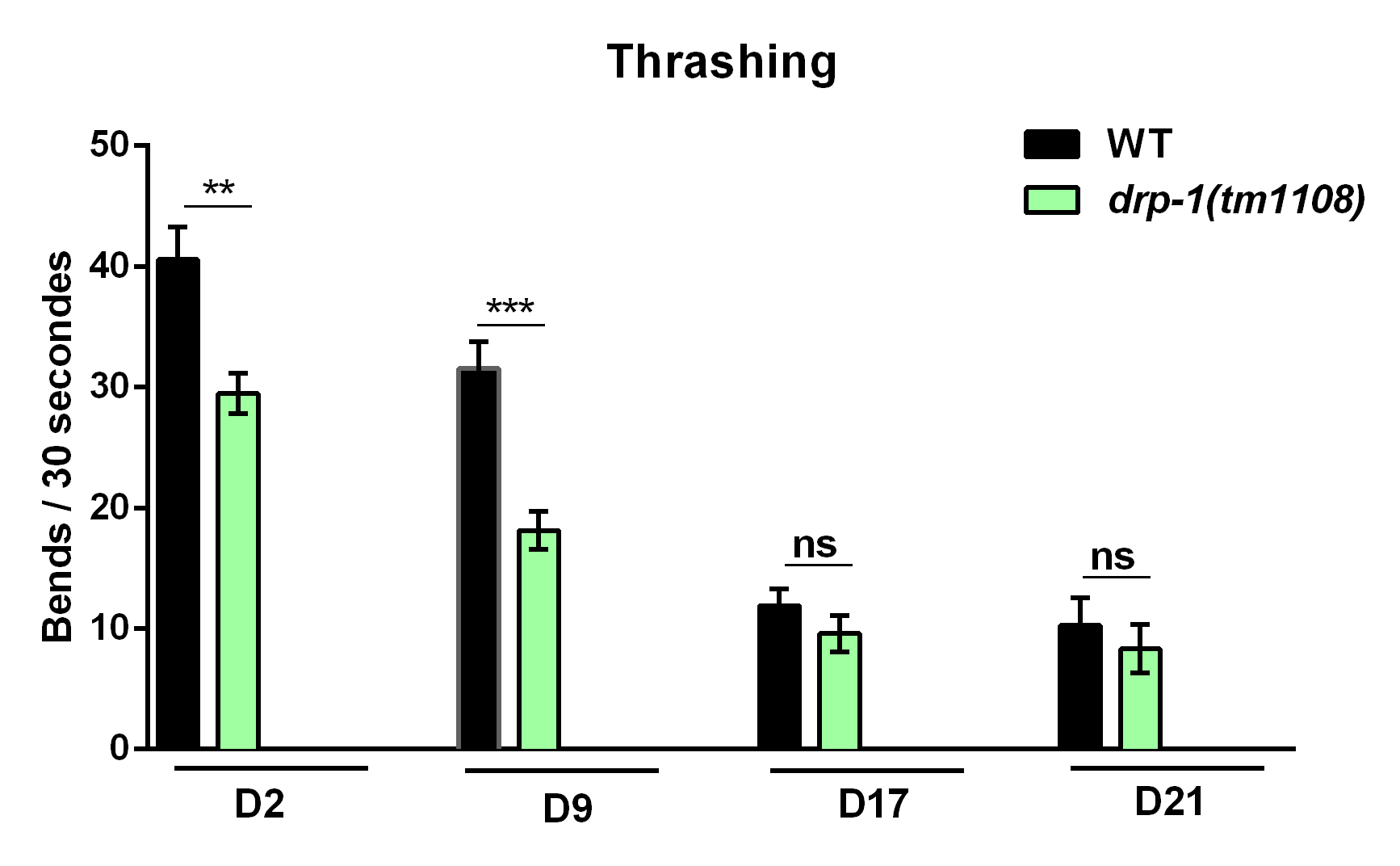
**

**S5 Fig. Effects of the absence of DRP-1 on worm locomotion over aging.**

Quantification of worm trashing in wild-type and in *drp-1(tm1108)* mutant worms at day 2, 9, 17 and 21 of adulthood of the nematode (n=25 worms at least). Data represent the mean obtained by pooling at least three independent assays. Errors bars represent SEM. Student test. ** p < 0.01, *** p < 0.001, n.s. indicates that the mean is not statistically significantly different from the mean obtained in the wild-type condition.


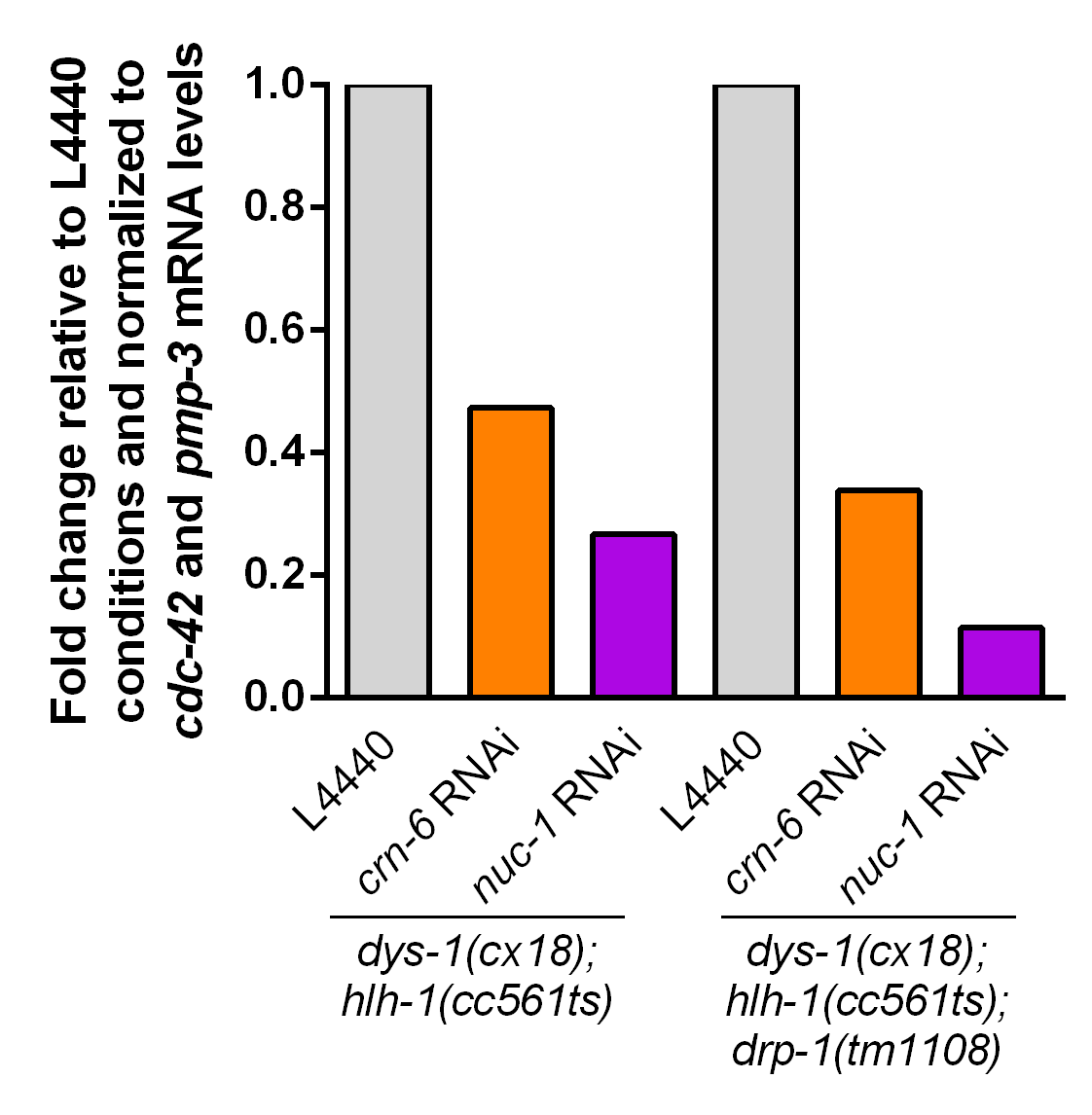


**S6 Fig. *crn-6* and *nuc-1* mRNA expression levels in *dys-1(cx18);hlh-1(cc561ts)* and in *dys-1(cx18);hlh-1(cc561ts);drp-1(tm1108)* mutant worms.**

The normalized mRNA levels in *dys-1(cx18);hlh-1(cc561ts)* and in *dys-1(cx18);hlh-1(cc561ts);drp-1(tm1108)* mutant worms fed with the empty vector L4440 were set as 1.

**S1 Protocol. Mitochondrial morphology analysis.** Gravid adults carrying the integrated *ccIs4251* transgene were allowed to lay eggs on NGM plates seeded with OP50 or HT115 containing RNAi constructs of interest or the empty vector L4440 for 6 hours before being removed from the plate. L4+3 day-old progeny worms were immobilized in 3.3mM levamisole on a 2% agarose pad for acquisition. Confocal images of the vulva area of lived paralyzed worms were taken on a Zeiss LSM 510 Meta or Zeiss LSM 800 using a 63X oil objective. *Fiji* software was used to process and to analyze captured images. To fix threshold, the inverse Fourier transformation (Inverse FFT) was used in order to minimize the noise and to sharpen the contrast between mitochondria and the background. Nucleus of each image was removed by hand (with the unaided human eye). « Analyze particles » command was used to obtain values for average size, aspect ratio (calculated by major axis / minor axis ; aspect ratio at 1 refers to a perfect circle) and elongation (calculated by 1-(minor axis / major axis ; 0 refers to no elongation) that reflects the “length-to-width ratio”, and form factor (calculated by perimeter² / 4πArea ; 0 refers to no branching) that reflects the complexity and branching aspect of mitochondria, for each mitochondrion in an image, ignoring mitochondria <5 square-pixels or on the edge of the image.

**S2 Protocol.** **RNA Isolation and Quantitative Reverse Transcription PCR (qRT-PCR)**

Synchronized population of ∼1,000 eggs of *dys-1(cx18);hlh-1(cc561ts)* or *dys-1(cx18);hlh-1(cc561ts);drp-1(tm1108*) mutant worms were grown on empty vector L4440 or on *crn-6* or *nuc-1* RNAi to late L4 staged worms at 15°C and harvested. Total RNA was isolated using Tris-reagent (Invitrogen) and DNase I (Invitrogen) was used to eliminate DNA contamination. cDNAs were synthesized using iScript cDNA Synthesis Kit (BIO-RAD). qRT-PCR reactions were performed using iQ SYBR Green Supermix (BIO-RAD) and the CFX Connect machine (BIO-RAD). Melting curve analysis was performed for each primer set to ensure the specificity of the amplified product. *pmp-3* and *cdc-42* were used as the internal controls so that the RNA level of each gene of interest was normalized to the levels of *pmp-3* and *cdc-42*. The quantative PCR primer sequences were for *pmp-3* : 5’GTTCCCGTGTTCATCACTCAT3’ and 5’ACACCGTCGAGAAGCTGTAGA3’ ; for *cdc-42*: 5’CTGCTGGACAGGAAGATTACG3’ and 5’CTCGGACATTCTCGAATGAAG3’ ; for *nuc-1*: 5’GCAAGGATCAGTCGGGAAAC3’ and 5’AGTACCATGCAACTCCACCT3’  ; for *crn-6*: 5’GGAACAATGTGCATCTACGATCA3’ and 5’CGGCTGAAAAGGTAGATGCG3’.
